# Anisotropic and self-healing hydrogels with multi-responsive actuating capability

**DOI:** 10.1038/s41467-019-10243-8

**Published:** 2019-05-17

**Authors:** Haili Qin, Tan Zhang, Na Li, Huai-Ping Cong, Shu-Hong Yu

**Affiliations:** 1 Anhui Province Key Laboratory of Advanced Catalytic Materials and Reaction Engineering, School of Chemistry and Chemical Engineering, Hefei University of Technology, 230009 Hefei, China; 2 Division of Nanomaterials and Chemistry, Hefei National Research Center for Physical Sciences at Microscale, Collaborative Innovation Center of Suzhou Nano Science and Technology, Department of Chemistry, University of Science and Technology of China, 230026 Hefei, China

**Keywords:** Gels and hydrogels, Self-assembly, Composites, Structural properties

## Abstract

Inspired by smart biological tissues, artificial muscle-like actuators offer fascinating prospects due to their distinctive shape transformation and self-healing function under external stimuli. However, further practical application is hindered by the lack of simple and general routes to fabricate ingenious soft materials with anisotropic responsiveness. Here, we describe a general in situ polymerization strategy for the fabrication of anisotropic hydrogels composed of highly-ordered lamellar network crosslinked by the metal nanostructure assemblies, accompanied with remarkably anisotropic performances on mechanical, optical, de-swelling and swelling behaviors. Owing to the dynamic thiolate-metal coordination as healing motifs, the composites exhibit rapid and efficient multi-responsive self-healing performance under NIR irradiation and low pH condition. Dependent on well-defined anisotropic structures, the hydrogel presents controllable solvent-responsive mechanical actuating performance. Impressively, the integrated device through a healing-induced assembly way can deliver more complicated, elaborate forms of actuation, demonstrating its great potentials as superior soft actuators like smart robots.

## Abstract (web summary)

The development of artificial muscle-like actuators is often hampered by the lack of general fabrication routes towards anisotropic responsive materials. Here, the authors fabricate anisotropic hydrogels by an in-situ polymerization strategy of a lamellar network, crosslinked by metal nanostructure assemblies.

## Introduction

For the sake of smart functions and movements, most tissues in living organism usually possess anisotropic morphology and structure from molecular to macroscopic levels^1–3^. For example, collagen fibrils closely packed in parallel arrays enable muscles with remarkable mechanical strength and one-dimensional contraction^4^. Bearing great similarities to biological systems, hydrogels with soft and wet feature have been widely explored as polymer scaffold for the engineering of man-made organs or tissues^5,6^. However, most synthetic gels demonstrate isotropic structures from uniform chain-growth process, leading to non-oriented deformation in response to environmental stimuli^7–9^. As such, it is of great significance but still challenging to fabricate anisotropic hydrogels and explore their distinctive behaviors arising from anisotropic effects as soft actuators.

Although anisotropic hydrogels with oriented structure and anisotropic responsiveness have been reported through the strategy of supramolecular self-assembly^10–14^, poor mechanical strengths are delivered resulting from weak crosslinks or polydispersed crosslinking points. In sharp contrast, the biological tissues are far more sophisticated than artificial soft-matters in all respects. Especially, mechanical properties, including elasticity, tensile stress at large deformation, and tear resistance for the notched region, play crucial roles in performing the functions of biological tissues^2,15^. On top of that, the ability of self-healing when damaged is another attractive characteristic widely existed in the living organisms, tightly related to their survivability and practicability^16–18^. Based on these aspects, it is urgently desired to develop ingenious strategies for the fabrication of artificial soft materials with anisotropic structures as well as multiple functionalities analogous to organisms.

With these considerations in mind and inspiration of nanocomposite hydrogels^19–22^, in case of serving flexible nanostructured assemblies as multifunctional crosslinkers bonded compactly with the polymer chains, the gels with well-oriented network structure and unique mechanical property are expected to be constructed^23^. For the sake of defect healing, strong and dynamic interaction between polymer chain and crosslinking point is required. Recently, the coordination interaction with tunable thermodynamics and large kinetic constant has been widely employed as effective healing motifs for smart supramolecular polymers^24–26^. To our knowledge, most of these reports are involved in the interactions between metal ions and ligating-atom-ended polymer chains with limited branches. Very lately, we proposed dynamic thiolate-Au (RS-Au) coordination from gold nanoparticles (NPs) as an efficient crosslinking mode for tough and self-healing gels^22^. Also, similar modes have been extended to other precious metals, such as silver, for the construction of functional composites^27,28^. Combined with easy-assembly feature of precious metal nanostructures when exposed to external stimuli, unique metal nano-assemblies would provide numerous possibilities in the creation of soft materials with anisotropic structures^29,30^.

Herein, a general, controllable strategy has been developed to fabricate a kind of anisotropic nanocomposite hydrogels composed of highly ordered lamellar precious-metal nanostructure assemblies (e.g. Ag, Au, Pt, and Cu) through the self-assembly of thiolate-modified metal nanostructures as highly branched crosslinkers for in situ polymerization. As a typical demonstration, in addition to the tough strength, the silver NP/polyacrylamide (PAM) (SNPP) hydrogel delivers an impressively anisotropic mechanical performance with the tangent modulus parallel to the lamellae 3.9 times the perpendicular direction arising from the entanglement of PAM network around the lamellar silver assembly architectures via RS-Ag coordination. Additionally, remarkable anisotropy in optics and swelling/de-swelling deformations is also observed. Thanks to the dynamic RS-Ag interactions as healing motifs, the hydrogel demonstrates a rapid and efficient multi-responsive self-healing performance induced by NIR laser and acid condition. With the combined merits of lamellar structure and anisotropic property, the composite exhibits controllable actuating performances in polar organic solvents in a structure-dependent way. Excitingly, the complicated actuation behaviors, such as robotic-arm lifting and hand grasping, can also be achieved by the healing-induced integration of hydrogel pieces with varied structures, promising its great potentials as soft actuators.

## Results

### Preparation of anisotropic SNPP hydrogels

**Fig. 1:**
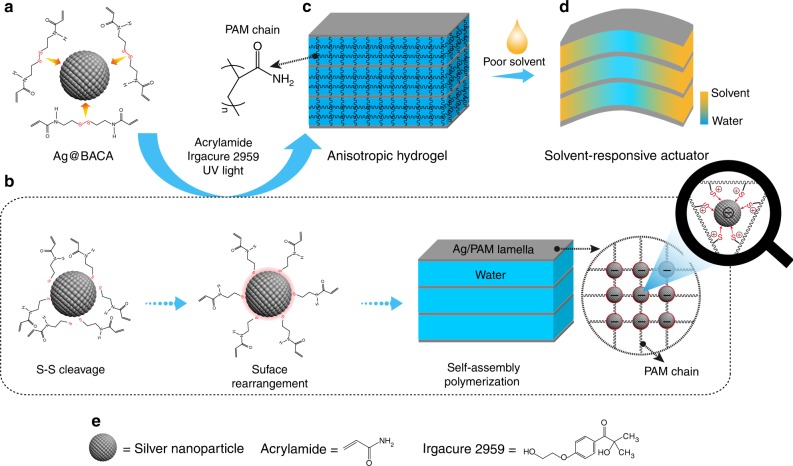
Schematic illustrations of the formation of SNPP hydrogel. **a** Ag@BACA nanocomposite linked by the RS-Ag coordination bonds. **b** When the BACA molecules were adsorbed onto the silver NPs, the S–S cleavage occurred. Under UV light, the cleaved BACA molecules on the surface of silver NPs were rearranged. With the help of the hydrophobically lamellar assembly of the Ag@BACA nanocomposites, the cofacially arranged Ag/PAM lamellae were formed by a UV light induced polymerization. **c** Upon prolonging the UV irradiation, the anisotropic hydrogel was fabricated by propagating the PAM chains around the Ag/PAM lamellae. **d** The anisotropic hydrogel shows actuating ability when exposed in poor solvent of PAM chains. **e** Molecular structure of the used co-monomers

The procedure for the fabrication of unique SNPP hydrogels is simple and controllable as schematically illustrated in Fig. 1. For typical experiments, silver NPs with an average diameter of 16 ± 2 nm were employed (Supplementary Fig. 1). The highly active silver atoms on the surface of silver NPs had strong affinity to sulfur-containing organic molecules in the form of RS-Ag coordination interaction (Fig. 1a)^31^. Here, a water-soluble surface ligand, *N*,*N*-bis(acryloyl)cystamine (BACA) comprised of disulfide bond in the molecular structure, was employed for the modification of silver NPs. In this way, the Ag@BACA nanocomposites were obtained with mixing BACA into the silver NP aqueous solution for few minutes at room temperature. Notably, when the BACA molecule was adsorbed onto the silver NPs, its disulfide bond was cleaved^32^, as revealed from the disappeared S–S stretch in the surface enhanced Raman spectrum (SERS) of Ag@BACA nanocomposites (Fig. 1b and Supplementary Fig. 2). Transmission electron microscopy (TEM) and UV-vis spectroscopy were used to study the binding behavior of BACA with silver NPs. TEM image and the corresponding element mappings showed the uniform distribution of BACA on the surface of silver NPs (Supplementary Fig. 3a–c). In the UV-vis spectra, owing to the active localized surface plasmon resonance (LSPR) effect of silver NPs^33^, the absorption peak of the Ag@BACA nanocomposite was red-shifted to 411 nm compared with the bare silver NPs (LSPR peak: 405 nm) (Supplementary Fig. 4a). Furthermore, the XPS spectrum of the composite with the characteristic peaks at 368.3 eV (Ag 3d_5/2_), 374.5 eV (Ag 3d_3/2_), and 163.5 eV (S 2p) confirmed the adsorption of BACA onto the silver NPs (Supplementary Fig. 4b). Under the UV irradiation (365 nm), the cleaved BACA molecules on the surface of the silver NPs were rearranged to minimize their surface energy arising from the photothermal effect of silver NPs and dynamic nature of RS-Ag bonds at a high temperature, which enabled the Ag@BACA nanocomposite hydrophobic (Supplementary Fig. 5). Under the solvophobic effect in the aqueous solution, the Ag@BACA nanocomposites were integrated into large 2D lamellar AgNP assemblies in single-nanoparticle-thickness (Supplementary Fig. 6a). For the Ag@BACA composites linked by the RS-Ag coordination, the silver NPs were acted as electron receptors, which made the AgNP lamellae have the high-density negative charges (Fig. 1b and Supplementary Fig. 6b). Under the effect of electrostatic repulsions, the AgNP lamellae were arranged in a cofacial way, producing a highly ordered lamellar network.

With the help of the hydrophobically lamellar assembly characteristic of the Ag@BACA nanocomposites, the Ag/PAM lamellae were formed by a polymerization process with the presence of the acrylamide as the monomer and Irgacure 2959 as the photo initiator during the early stage of the UV irradiation (Supplementary Fig. 7a). The FT-IR spectrum of the Ag/PAM lamellae showed the greatly reduced intensity of C = C vibration at 1667 cm^−1^ compared with that of Ag@BACA nanocomposites, which confirmed the polymerization between the acrylamide and the modified silver NPs (Supplementary Fig. 7b). Under the synergistic effect of the cofacial arrangement of the Ag/PAM lamellae with the abundant vinyl groups existing on the lamella surfaces, a 3D lamellar assembly network was constructed and acted as the large nano-crosslinker. Upon prolonging the polymerization time, the anisotropic hydrogel consisting of the 2D AgNP assembly lamellae and the entangled polymer network was fabricated by continuously propagating the PAM chains and increasing the density of the polymer network among the lamellae (Fig. 1c). This unique structure makes the anisotropic hydrogel promising potential as an actuator driven by external stimuli, such as poor solvent (Fig. 1d).

**Fig. 2:**
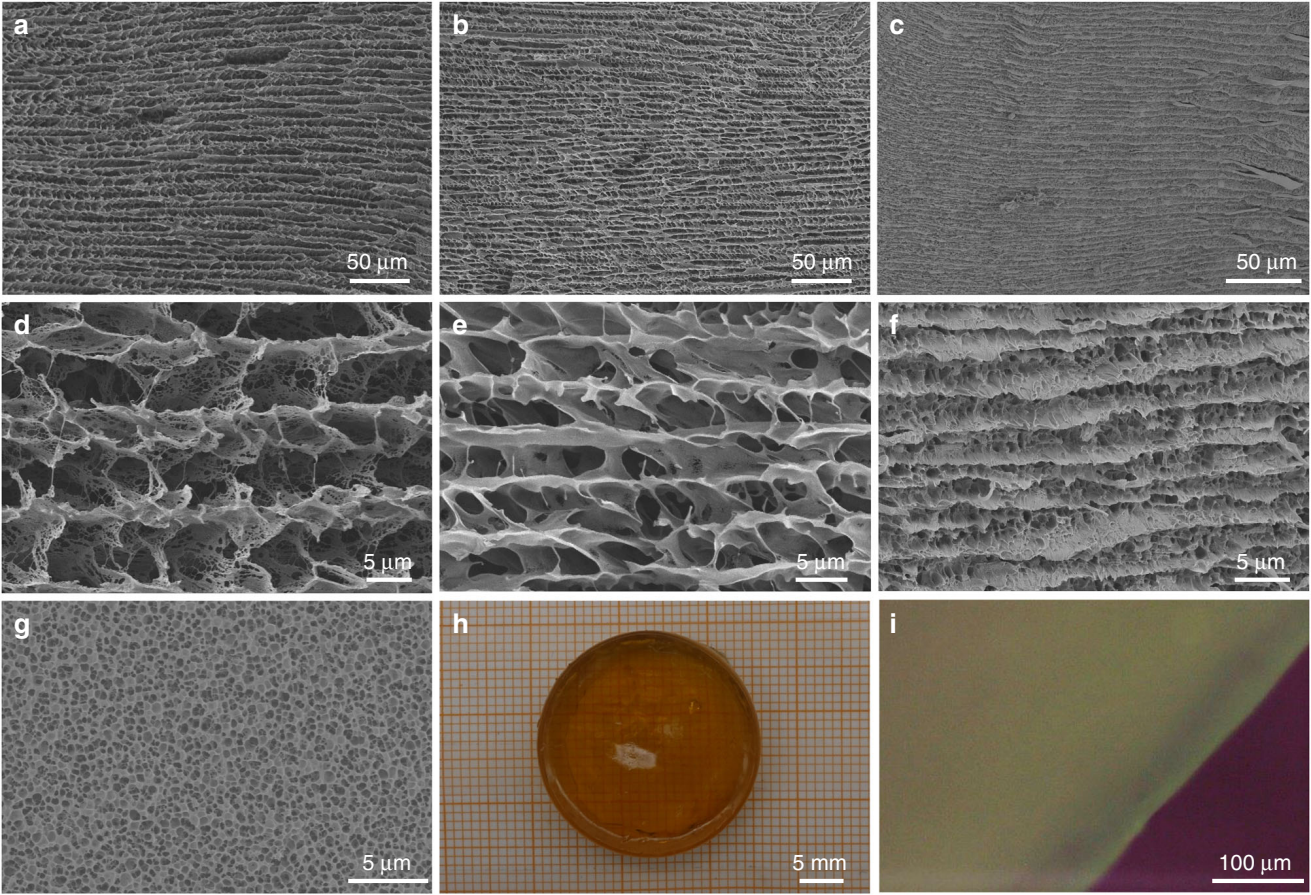
Preparation of anisotropic SNPP hydrogels. Low and high magnification SEM images of time-dependent network structure evolution in the polymerization process: **a**, **d** 5 min, **b**, **e** 10 min, and **c**, **f** 30 min. **g** SEM image of the surface of gel network. **h** Optical image of the hydrogel. **i** POM image of the gel viewed parallel to lamellar structure under cross-polarized light

Time-dependent morphology evolution of the anisotropic hydrogel was studied to track the formation process. At the beginning (UV irradiation for 1–2 min), the mixture solution became viscous, and the large 2D Ag/PAM lamellae were found in the solution as seen from the TEM image (Supplementary Fig. 7a). As the irradiation proceeded for 5 min, the reaction system changed from sol to gel. Corresponding freeze-dried SEM images obviously showed the lamellar structures in a parallel alignment with a large interlamellar spacing of about 8–10 μm (Fig. 2a, d). With the UV irradiation for 10 min, a dense and compact polymer network was obtained between the lamellae, and the interlamellar spacing was decreased to 2–4 μm (Fig. 2b, e). Eventually, the SNPP hydrogel with a bright and homogeneous color was fabricated after the UV irradiation for 30 min followed with the lamellae stacked in a close and highly ordered way in large areas (Fig. 2c, f, h). The magnified SEM image of the upper surface of the hydrogel showed a uniform and dense network, quite different from the layered structure of its cross-sectional morphology (Fig. 2g). Based on these analyses, the SNPP hydrogel was prepared by producing the Ag/PAM lamellar framework based on the 2D assembly of the modified silver NPs and subsequently increasing the density of the entangled polymer network by propagating PAM chains through a UV light induced polymerization.

The element mappings of the freeze-dried gel presented the homogeneous dispersions of Ag, C and S elements, indicating the uniformity of silver NPs in the network (Supplementary Fig. 8). TEM images of the freeze-dried hydrogel pieces by ultrasonic treatment in the ethanol showed a uniform distribution of the silver NPs in the entire hydrogel (Supplementary Fig. 9a). Furthermore, the defocused contrast of silver NPs in the high-magnification TEM image provided an evidence for the lamellar alignment of the silver assemblies (Supplementary Fig. 9b). Notably, the network in the hydrogel was well-oriented, as revealed by polarizing optical microscope (POM) measurement^10,34^. Gel samples were cut perpendicularly to AgNP lamella for test. The POM image with bright and uniform color fully proved the highly aligned structures throughout the gels (Fig. 2i). To confirm the lamellar structures, excessive potassium peroxydisulfate (KPS) was used for removal of 2D silver aggregates^22^. The color of hydrogel gradually fading to colorless indicated the complete removal of Ag NPs (Supplementary Fig. 10). Without silver crosslinks, the faded hydrogel was composed of plenty of porous polymer sheets in a parallel arrangement (Supplementary Figs. 11 and 12), quite consistent with our statement above.

### Multiple anisotropic behaviors

**Fig. 3:**
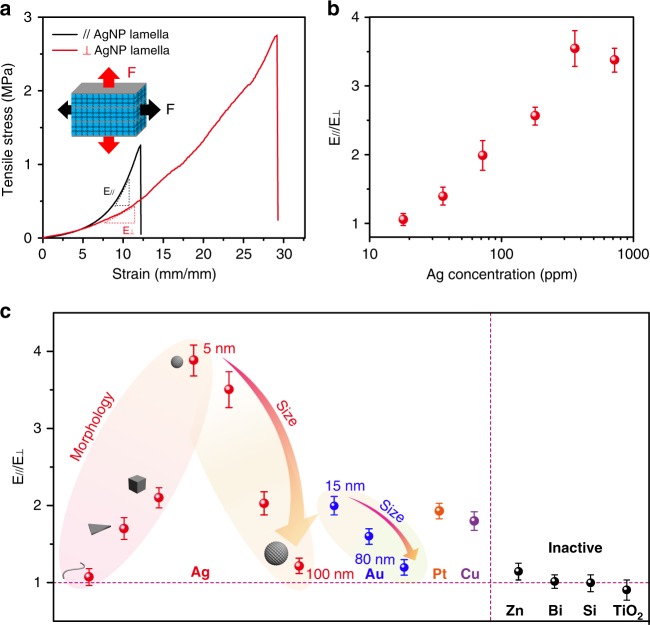
Anisotropically mechanical behavior. **a** Tensile stress–strain curves of SNPP hydrogel with stress applied parallel and perpendicular to the AgNP lamella. **b** Tangent modulus ratio (E_∥_/E_⊥_) calculated from tensile stress-strain curves of SNPP hydrogels, plotted against the content of silver NPs. **c** E_∥_/E_⊥_ of hydrogels fabricated by nanostructures with varying sizes, morphologies, and compositions. Error bars show the SD with sample size of 3

The mechanical property of the obtained SNPP hydrogel was systematically investigated. As shown in the photograph (Supplementary Fig. 13a), a hydrogel piece with the length of ~1 cm was stretched to nearly 20 cm without any rupture or crack, indicating its excellent stretchable behavior. In the following, a tensile machine was employed to study the anisotropic response on mechanical performance with the stress applied in the orthogonal directions. The mechanical behaviors of hydrogels containing the lamellar network structures were tightly dependent on the direction of the applied stress (Fig. 3a). In the initial stage of the tensile stress–stain curves (Supplementary Fig. 14), the Young’s modulus of the hydrogel piece stretched in the perpendicular direction of AgNP lamella (87 kPa) was higher than that in the parallel direction (25 kPa). However, during the continuously stretching process, the hydrogel exhibited prominent anisotropic mechanical performance in two directions. For example, the stretch parallel to the AgNP lamella yielded a tangent modulus E_∥_ of 221 kPa in the strain regions of 7–12, and a critical stretch of 2921% at rupture with the tensile stress at 2.76 MPa, which was 3.9 times the tangent modulus E_⊥_ of 57 kPa with the tensile stress of 895 kPa at a ultimate stretch of 1217% under a perpendicular stretch. In contrast, the conventional hydrogels without silver NPs crosslinked showed the isotropic characteristics with a weak stress of 0.98 MPa at an elongation of 1650% when stretched in orthogonal directions, and also no anisotropy was found in morphology analysis (Supplementary Fig. 15).

This distinctively mechanical anisotropy was ascribed to the anisotropic structures composed of the well-oriented AgNP assembly lamellae entangling with flexible PAM network^35,36^. In the perpendicular direction of AgNP lamella, the high modulus of the hydrogel in the initial stretching was due to the break of the well-oriented AgNP assembly lamellae. Subsequently, the gradual unzipping of the PAM chains in the hydrogel network resulted in a modulus increase with a smooth slope and a large elongation. When stretching the hydrogel piece in the parallel direction of AgNP lamella, the twisted PAM chains entangling in the anisotropic AgNP lamellae were stretched in the initial process, which contributed to a low Young’s modulus. The subsequent stretching led to the slipping and collapse of the AgNP lamellae in the hydrogel, which resulted in a sharply increased mechanical strength and a low elongation. Additionally, the influence of the concentration of silver NPs in the network on the mechanical anisotropy was systematically studied (Supplementary Fig. 16). Statistics suggested that the tangent modulus ratios of the hydrogels between the two directions (E_∥_/E_⊥_) were increased when improving the silver NP content in the gels (Fig. 3b). The higher degree of mechanical anisotropy was attributed from the formation of more obvious AgNP assembly lamellae in the initial polymerization and the resulting higher-orientation structure of the hydrogel with increasing the contents of silver NPs (Supplementary Fig. 17). However, an over high content of silver NPs would decrease the mechanical anisotropy because of the deteriorated consistency of the assembly nanostructures in the polymer network^35^.

To understand the key roles of the 2D nano-assemblies in the formation of anisotropic structures, the other 15 nanostructures with different morphologies, sizes and compositions were employed (Supplementary Fig. 18), and the corresponding anisotropic analysis was summarized in Fig. 3c based on the E_∥_/E_⊥_ values calculated from the tensile stress–strain curves (Supplementary Figs. 19–21). It was investigated that a distinctively mechanical anisotropy was detected in the precious metals, such as Au, Ag, Pt, and Cu, resulting from their unique coordination interactions with sulfur-containing BACA molecules as well as the surface-induced S–S bond cleavage (Supplementary Fig. 22)^37–39^, and the metals, nonmetals and metal oxides without these features were inactive. Notably, with decreasing the size of nanoparticles, the anisotropy was more prominent correspondingly as confirmed by 5–100 nm of Ag NPs and 15–80 nm Au NPs. It was also found that the morphology of nanostructures had remarkable influences on the formation of anisotropic structure. For example, with improving the aspect ratio of Ag nanostructures from nanoparticle, nanocube, nanoplate to nanowire, the anisotropic performance was obviously-deteriorated, possibly due to the weaker assembly in the lamellae. SEM images confirmed that the hydrogels fabricated from the precious metals with small aspect-ratio morphology and small particle size exhibited more prominent anisotropic structures (Supplementary Fig. 23). Based on above systematic study, a general and controllable strategy was presented for the fabrication of anisotropic hydrogels relied on the morphologies, sizes, and compositions of the used nanostructures.

**Fig. 4:**
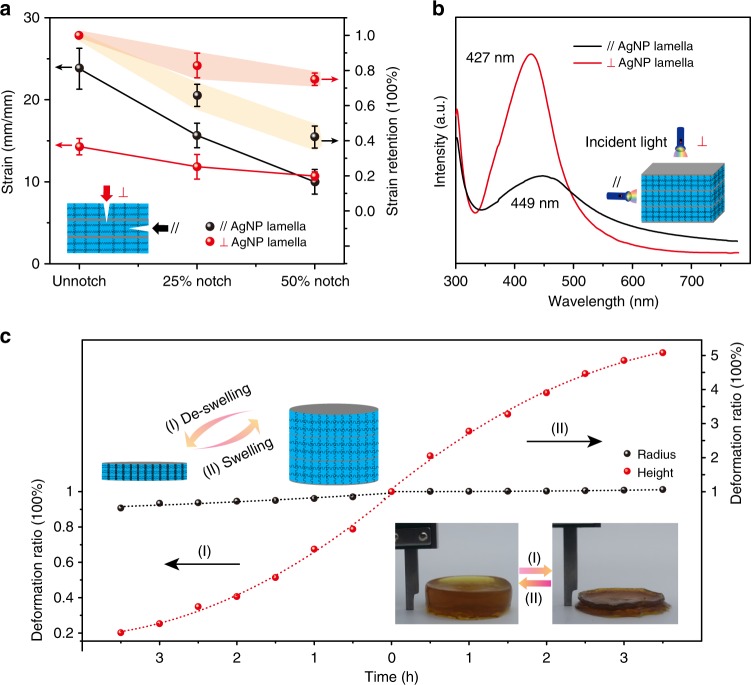
Anisotropy on notch-insensitive, optical, and swelling performances. **a** Ball plot of SNPP hydrogel with notches parallel and perpendicular to AgNP lamella and notch ratios from 0 to 50%. Error bars show the SD with sample size of 3. **b** UV-vis spectra of SNPP hydrogel with the incident light from two orthogonal directions. **c** Deformation ratio profiles of radius and height of hydrogel against time during the de-swelling and swelling process. The inset images show the cycling between the initial and de-swollen states

Furthermore, the highly branched silver crosslinks enabled the polymer network notably notch-insensitive. For typical demonstration, hydrogel pieces with different proportions of notches perpendicular and parallel to AgNP lamellae varying from 25 to 50% of their original width were performed on the tensile machine, respectively (Supplementary Figs. 24 and 25). The mechanical strength and elongation at critical stretch of these notched gels and pristine samples were summarized (Fig. 4a). It was clearly observed that the SNPP hydrogel exhibited exceptional notch-insensitive performance when the notch perpendicular to the AgNP lamella, otherwise, great crack propagation was occurred when the notch along the lamella, revealing a great anisotropy.

In order to investigate the optical anisotropy of the SNPP gel, UV-vis spectra were recorded with the incident light across and along the AgNP lamella (Fig. 4b). Specific assembly behavior of silver NPs and the oriented arrangement of lamellae enabled the hydrogel to show anisotropic optical property. As such, the plasmonic resonances of 427 and 449 nm with distinct intensity were detected when the incident light perpendicular and parallel to the AgNP lamellae, respectively, quite consistent with the structural characteristics discussed above.

Besides the anisotropically mechanical performance and optical property, the SNPP hydrogel also performed distinctly anisotropic property in the de-swelling and swelling processes. The de-swelling and swelling performances of the gels were typically observed by placing the gel cylinders in an oven at 60 ^o^C and then immersing the gels in water at room temperature, followed with monitoring their height and radius changes as a function of time. The initial size of the gel used for tests was 11.65 mm of radius by 4.32 mm of height determined by Vernier caliper. The relevant data was recorded once every half an hour (Fig. 4c and Supplementary Fig. 26). During the de-swelling process in the left part of Fig. 4c, it was worth noting that the change of the gel radius was negligible in 3.5 h. However, the obvious height changes occurred, especially in the initial 2 h, with nearly 80% shrunk and the final height was only 0.78 mm. When swollen again, the shape deformation mainly took place in height rather than radius. A nearly 410% of height increase was investigated, followed with less than 7% expansion on the radius. Additionally, the freeze-dried SEM image of the shrunk SNPP gel was made to further reveal the anisotropic structure of gel network (Supplementary Fig. 27b). After the de-swelling process, the lamellae became much denser and more ordered, which was proved by the POM image with bright color (Supplementary Fig. 27c), quite agreeing with great change in the thickness of the gel. In contrast, the conventional hydrogel with physically incorporated silver NPs showed isotropic feature with large deformations in both the thickness and radius during de-swelling and swelling processes (Supplementary Fig. 28). The analyses above fully demonstrated the SNPP hydrogel with unique high-level one-dimensional swelling and de-swelling resulting from their unidirectional alignment of lamellar network.

### Multi-responsive self-healing performance

**Fig. 5:**
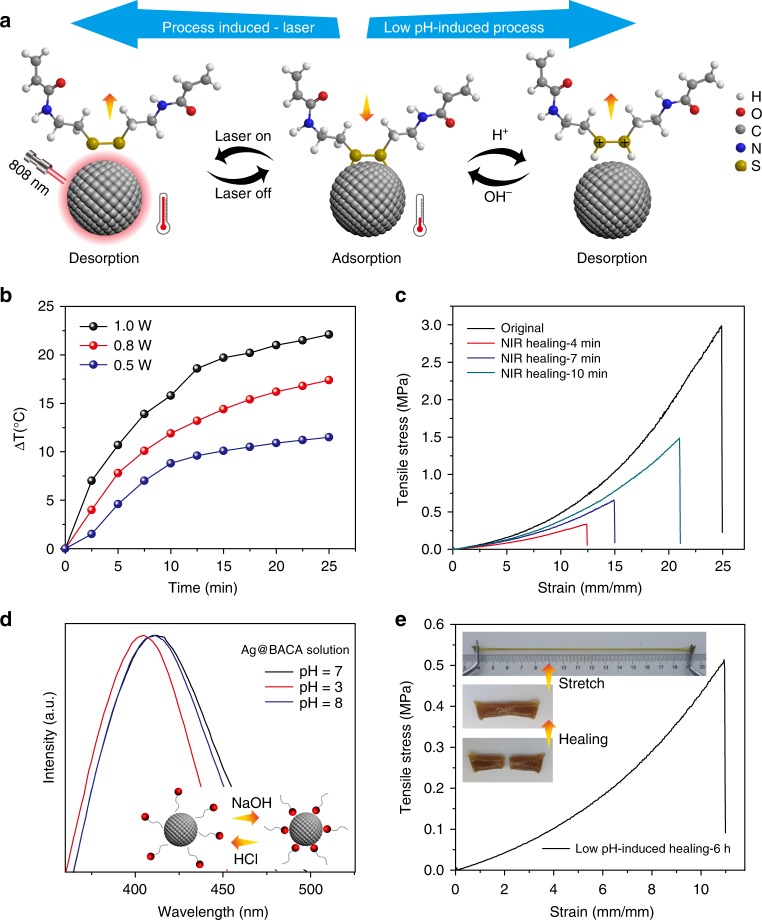
Self-healing performance. **a** Schematic illustration of NIR laser-induced and pH-mediated self-healing mechanisms. **b** Time-dependent temperature changes of the SNPP hydrogel when exposed to NIR laser with various output power. **c** Tensile stress–strain curves of hydrogel piece relative to its original state and different healing times under NIR laser. **d** UV-vis spectra of Ag@BACA composite recorded to monitor the binding behavior between BACA and silver NPs in different pH solutions. The inset schemes illustrate the reversible binding of BACA and silver NPs with the regulation of pH. **e** Tensile stress–strain curves of the healed SNPP hydrogel piece through a pH mediated way. The inset optical images show high stretchability of the healed gel induced by the acid solution

Similar to biological tissues, the SNPP hydrogels with anisotropic network structure demonstrated a rapid and efficient self-healing performance since they contained large amounts of dynamic and reversible coordination interactions of RS-Ag from the Ag@BACA composites in the network. The particular crosslinks consisting of the silver NPs attached with disulfide bond-ended polymer chains as ligands were regulated when exposed upon the external stimuli, such as light and pH (Fig. 5a). It is a general agreement that high temperature can trigger the surface ligand switching on/off from the solid surface^40^. Accordingly, by means of exceptional photothermal behaviors of silver NPs (Fig. 5b)^41^, the SNPP hydrogel was self-healable under the stimulus of NIR laser owing to the RS-Ag induced surface reconstruction. The typical experiment was conducted on two individual gel pieces. With drawing the pieces into a close contact and exposing the incision under NIR laser for a few minutes, they merged together and could be stretched manually (Supplementary Fig. 29). SEM images exhibited a completely fused structure of the healed hydrogel (Supplementary Fig. 30). The mechanical behaviors of the healed samples were further evaluated by tensile strain–stress curves and the healing efficiency was defined as the strain ratio between the healed and original pieces. Prolonging the irradiation time, the healing efficiency was increased (Fig. 5c). With the irradiation of 10 min, a large stretch of 2100% was achieved with the efficiency as great as 85%. In addition, an increased healing efficiency was demonstrated when improving the content of silver NPs in the gel arising from the increased RS-Ag bonds in the network (Supplementary Fig. 31). In contrast, the hydrogel pieces without silver NPs (SNPP-0) could not be healed when exposed to NIR laser or in an oven (Supplementary Fig. 32a, b). Notably, even for SNPP hydrogel with high content of silver NPs, no healing was observed when placed in the oven (60 ^o^C) for a long time as a result of the poor thermal conductivity of the gel pieces (Supplementary Fig. 32c). These analyses fully revealed that dynamic RS-Ag bonds combined with exceptional photothermal property of noble metal NPs enabled the obtained hydrogels superiority in self-healability compared with previously reported hydrogels (Supplementary Table 1)^22^.

In addition to the laser-induced self-healing process, the dynamic silver crosslinks inside the hydrogel network can also be healed through a pH-mediated way. Such healing capability was attributed to surface reconstruction of the fractured parts based on the rupture of RS-Ag bonds in SNPP hydrogel (Fig. 5a). From the viewpoint of Lewis theory of acids and bases, sulfur atom-ended polymer chains tended to bind with protons from strong acid rather than silver atoms^28^. UV-vis spectra were carried out to monitor the reversible binding behavior between BACA and silver NPs through controlling pH values of Ag@BACA composite solutions. In acid condition, the LSPR peak of the nanocomposites was shifted from 411 nm to 405 nm, assigned to the peak of uncovered silver NPs (Fig. 5d), revealing the detachment of BACA from the silver NP surface. When adjusting pH value of the above solution to 8.0 with NaOH to guarantee the complete removal of the introduced protons, the LSPR peak was back to 411 nm correspondingly, indicating the recombination of BACA and silver NPs. These analyses fully demonstrated dynamic, reversible nature of RS-Ag bonds in SNPP hydrogel in acid solution, providing a gentle way to heal the fractured materials. A typical self-healing experiment on the gels was conducted by brushing the end of two individual SNPP gel pieces with acid solution (1 mol L^−1^ HCl) for surface activation and then bringing in contact with each other for healing. Combined with the swelling performance of the hydrogel, the acid solution would be permeated into the hydrogel piece continuously, leading to an increase of pH value at the interface (Supplementary Fig. 33). Impressively, after several hours, the healed gel could sustain large stretch under external load with an elongation of 1100% of its initial value as measured from the tensile stress-strain curve, suggesting an impressive healing effect (Fig. 5e). In contrast, SNPP-0 hydrogel pieces under the same self-healing procedure showed no healing behavior even for a long time (Supplementary Fig. 34). As a pH-responsive bond, RS-Ag endowed the developed tough and stretchable SNPP hydrogels with superior healing performance compared with other pH-responsive motifs, such as hydrogen bond (Supplementary Table 2).

### Anisotropically mechanical actuator

**Fig. 6:**
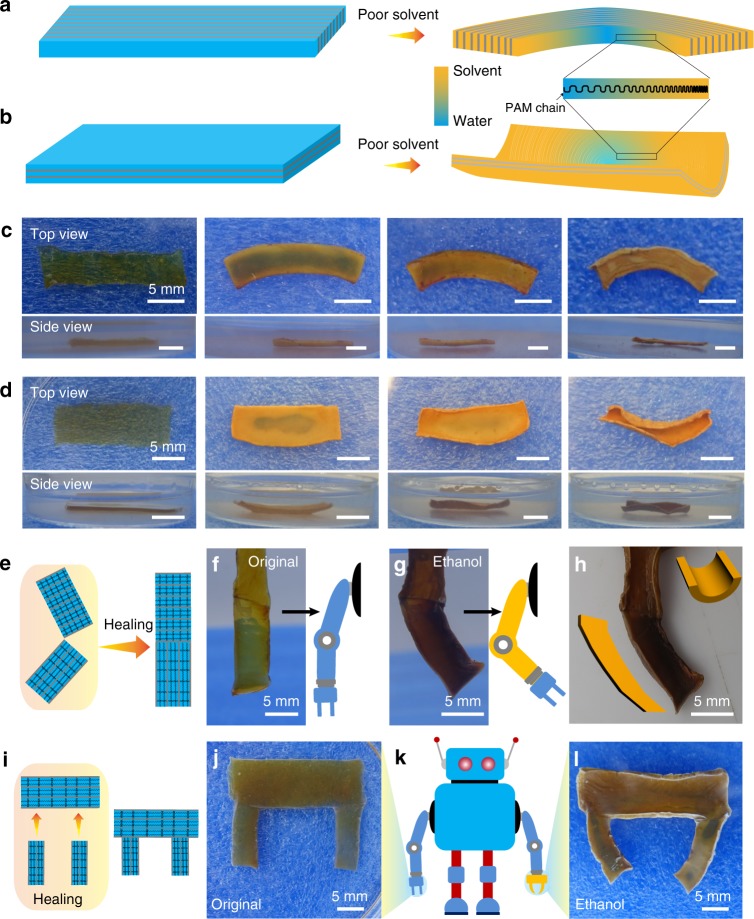
Solvent-responsive anisotropic actuating performance. **a**, **b** Schematic illustration of actuator mechanism for the SNPP gel piece when exposed to poor solvent. In situ real-time optical monitoring the bending process of gel piece with the lamellae perpendicular (**c**) and parallel (**d**) to the top surface when placed in ethanol, from top and side views, respectively. All the scale bars in **c**, **d** are 5 mm. **e** Schematic illustration of assembly of robotic arm by two hydrogel pieces with lamellae in different orientations through a healing procedure. **f**, **g** Combined with the anisotropic structures, the integrated hydrogel piece exhibits distinct actuations varied from the parts when placed in ethanol, just like a robotic arm with vertical lifting. **h** Further optical image proves the in-plane and out-of-plane deformations. **i** Schematic illustration of complicated assembly of robotic hand composed of two fingers and one palm and conducted by three hydrogel pieces with lamellae in different orientations through a healing procedure. **j**–**l** The integrated hand shows a grasping action when immersed in ethanol

The SNPP hydrogel pieces with densely lamellar polymer network in varying orientations exhibited an actuation when exposed to poor solvent stimulus of PAM. Owing to the poor solubility in ethanol, the polymer network in the lamellar structures would be greatly contracted. In this way, the actuation performance was behaved and determined by the orientation of lamellae in the gel piece. Especially, on the basis of the ordered lamellar stacking and anisotropic tangent modulus, gel pieces would perform as actuators in an anisotropic way during the permeation of poor solvent (e.g. ethanol) (Fig. 6a, b). Typically, when the gel piece composed of amounts of lamellae perpendicular to the surface was placed in ethanol, a gradual in-plane bending was easily observed (Fig. 6c). As for the gel piece with the lamellae parallel to the surface, an out-of-plane bending was delivered (Fig. 6d). For better understanding of the actuating process, the whole evolution process was monitored from both the top and side views. During the bending process, the transparent gel piece gradually became deep yellow from the edge to inside with the permeation of ethanol. The excellent anisotropic structure motivated the gel piece to show an incredible bending performance as a result of an oriented contraction without any external physical forces, and these actuating behaviors above were reversible when soaking into water again (Supplementary Fig. 35). It should be noteworthy that the bent hydrogel could be steady in the solvents for several days and became to be less stretchable and mechanically tougher in contrast to its original state because of the greatly contracted polymer network (Supplementary Fig. 36). For systematic study, the other 8 solvents with different polarities were also employed (Supplementary Fig. 37). It was found that the polar solvents with little solubility to PAM chains, that is poor solvent, such as acetone, methanol, dimethylformamide (DMF), and tetrahydrofuran (THF), could also trigger the gel pieces as actuators. Notably, the actuating behavior was controllable as shown from the largest bending deformation in ethanol due to its weakest solubility to PAM as quantitatively indicated from the lowest Gibbs free energy change per unit area (Δ*G* = −84 mJ m^−2^)^42^. However, inconspicuous deformation was observed when placing in the polar solvents with large solubility to PAM, such as dimethyl sulphoxide (DMSO) and ethylene glycol (EG), and non-polar solvents such as petroleum ether and cyclohexane.

Inspired by the structure-dependent actuation and self-healing ability, the complicated forms of actuation can be achieved on the integration of hydrogel pieces with lamellae in varied orientation through a healing procedure. As a demonstration, a soft device was integrated through healing two hydrogel pieces with different lamellar orientations, and then being immersed into ethanol (Fig. 6e–g). Several minutes later, the distinct actuations composed of both in-plane and out-of-plane deformations were found in the assembled hydrogel piece, showing a vertical lifting behavior like a robotic arm (Fig. 6h). With the healing-induced assembly strategy discussed above, more complicated actuations could be realized when more hydrogel pieces were integrated. Figure 6i–k showed that three pieces of gels were assembled to perform as a robotic hand composed of two fingers and one palm. When exposed to ethanol, it was observed that the hand-like soft actuator delivered a palm-bending-fingers-clamping behavior (Fig. 6l). Based on these analyses, such a healing-induced integration provided the SNPP hydrogel with the complicated and elaborate actions, indicating its potentials as superior actuating materials.

## Discussion

In summary, we have developed a general strategy to fabricate a kind of anisotropic hydrogels consisting of highly-ordered precious-metal nanostructure assembly lamellae through an in situ polymerization process by serving the thiolate-modified metal assemblies as multifunctional crosslinkers. Owing to the ordered lamellar structures in macroscopic scale, the typical SNPP hydrogels showed distinct anisotropy on mechanical properties, including tangent modulus and notch-insensitivity, and highly one-dimensional oriented swelling and de-swelling deformations. Combined with dynamic RS-Ag interaction as healing motifs and excellent photothermal property of silver NPs, the composite demonstrated remarkably multi-responsive self-healing capability under the irradiation of NIR laser and low pH condition. These prominent performances along with unique anisotropic structure made such gels to exhibit impressively in-plane and out-of-plane bending actuations in a structure-dependent way. Moreover, with the healing-induced programmed integration of composite pieces, the actuator devices performed more complicated, elaborate forms of actuation. The present strategy provides a way to rational design and controllable construction of high-performance ingenious soft materials with striking similarity to biological tissues, which would find great potential applications in artificial muscle and smart actuators.

## Methods

### Nanostructures

Ag NPs (5 nm), TiO_2_ NPs (20–40 nm), Bi NPs (20 nm), Zn NPs (40–60 nm), and Si NPs (10–30 nm) were purchased from Beijing Zhongkeleiming Daojin Technology Co. Ltd. Ag NPs (16 nm) and Cu NPs (5 nm) were purchased from Nanjing XFNANO Materials Tech Co. Ltd.

### Preparation of Ag NPs

Ag NPs (diameter: 25–35 nm): Briefly, 0.4 mmol soluble starches and 0.16 mmol l-lysine were premixed in deionized water (4 mL). Then, 4 mL of AgNO_3_ solution (0.2 mol L^−1^) was added into the above solution under magnetic stirring. The resulting solution was heated to 150 ^o^C in a closed Microwave Synthesizer. After 10 s, monodispersed Ag NPs with yellow–green color were obtained. The products were collected by centrifugation at 9000 rpm for 10 min and washed by deionized water for three times.

Ag NPs (diameter: 80–100 nm): 0.3 g PVP and 0.2 g AgNO_3_ were dissolved in EG (50 mL). Then, 135 μL KCl solution (7.5 mg mL^−1^) was added into the above mixture under magnetic stirring. Followed with heated to 130 ^o^C in an oil bath for 5 h, Ag NPs were obtained. Note: EG was refluxed for 5 h at 140 ^o^C prior to use and the KCl solution was prepared with the refluxed EG.

### Preparation of Ag nanocubes

In typical fabrication, EG was refluxed for 5 h at 140 ^o^C prior to use. Then, 12 mL EG was heated in a 50 mL glass vial at 150 ^o^C for 5 min, followed with adding 0.16 mL Na_2_S solution (0.234 mg mL^−1^). After 10 min, 3 mL PVP solution (20 mg mL^−1^) and 1 mL AgNO_3_ solution (48 mg mL^−1^) were sequentially added into above solution. When the solution was kept for 20 min and the color changed to green, Ag nanotubes were prepared. All solution used above were prepared with the refluxed EG.

### Preparation of Ag nanoplates

Before the synthesis of Ag nanoplate, Ag seeds were firstly prepared as follows: AgNO_3_ (0.11 mM) and Na_3_-citrate (2.05 mM) were mixed in 110 mL deionized water under magnetic stirring. Then, 3 mL NaBH_4_ aqueous solution (5 mM) was added into above solution under stirring for 10 min, and aged for 5 h before use. As for the synthesis of Ag nanoplates, 10 mL of AgNO_3_ solution (5 mM), 7.5 mL of PVP solution (0.7 mM), 7.5 mL of Na_3_-citrate solution (30 mM), and 62.5 mL of l-ascorbic acid (1 mM) were added into 6.4 mL of seed solution under magnetic stirring. The whole solution was kept for 15 min. Until the color was unchanged, Ag nanoplates were prepared.

### Preparation of Ag nanowires

In the typical synthesis, PVP (5.86 g) and glycerol (190 mL) were added into a three-necked round flask, followed with heating at 110 ^o^C until dissolved completely. When the above solution was cooled to room temperature, AgNO_3_ (1.58 g) and NaCl solution (59 mg of NaCl dissolved in mixture containing 10 mL of glycerol and 0.5 mL of deionized water) were sequentially added into above solution under gently stirring. Then, the solution was heated at 210 ^o^C for 30 min. With the color of solution changed from pale white to light brown, and eventually grayish-green, Ag nanowires were prepared.

### Preparation of Au NPs

Typically, Au NPs with diameter of 15 nm were prepared as follows: 50 mL of HAuCl_4_ solution (0.3 mM) was firstly heated at 100 ^o^C. Then, 1 mL of Na_3_-citrate (38.7 mM) was added into above solution. After 5 min, the color of solution changed to brilliant red and Au NPs were prepared. Au NPs with diameters of 40 and 80 nm were achieved by simply controlling the Na_3_-citrate/HAuCl_4_ precursor ratio at 2:1 and 0.7:1, respectively.

### Preparation of Pt NPs

Typically, 4 mL H_2_PtCl_6_ aqueous solution (7.72 mM) was mixed with 51.4 mg PVP, followed with diluted to 50 mL with the addition of deionized water. Then, 30 mL NaBH_4_ solution (15.5 mM) was dropwisely added into above mixture with the color changed to dark brown. The whole solution was kept stirring for 2 h and Pt NPs were obtained.

### Preparation of anisotropic hydrogels

Taking the preparation of the SNPP hydrogel as a typical example: Firstly, 2 mg of BACA was added into 5 mL of Ag NPs solution, followed with ultra-sonication for 10 min to ensure fine coating of BACA onto the surface of Ag NPs. Secondly, 200 mg mL^−1^of acrylamide as monomer, and 3 mg of Irgacure 2959 as photo-initiator were added into above solution under magnetic stirring. Then, the mixture was bubbled with N_2_ for 10 min to eliminate the O_2_ dissolved, followed with the degasification in a vacuum oven at room temperature. With the irradiation of the mixture under UV light (high-pressure mercury lamp, 365 nm, 300 W) for 30 min, the SNPP hydrogel with bright orange–yellow color was prepared. Other anisotropic hydrogels were prepared with varied nanostructures through a similar method.

### Self-healing procedures

The self-healing process was executed with two individual hydrogel pieces cut from a whole sample. The laser-induced process was carried out under the irradiation of NIR laser (808 nm, 1 W) with a spot area (1 cm × 0.5 cm). The contact interface between hydrogel pieces was placed at a distance of 10 cm from the NIR laser. The low pH-induced process was conducted with acid solution of HCl (1 mol L^−1^) brushed on these hydrogel surfaces before contact. The healing efficiency was investigated through the tensile experiments. For comparison, the control experiments were also performed without NIR laser or acid solution. The whole experiment was performed at room temperature.

### Actuating performances

The actuating process was carried out with two individual hydrogel pieces cut from the same sample but in orthogonal directions. Then, the gel pieces with the lamellae perpendicular and parallel to the top surface were separately and completely soaked in the poor solvent of PAM, for example, ethanol. The whole actuating process was monitored by camera from both top and side views with time. For a systematic investigation, other solvents with different polarities were also conducted for actuating study with the same procedure as ethanol.

### Materials characterization

SEM images were performed on a field-emission scanning electron microscope (Zeiss Merlin Compact). The samples for SEM investigation of the time-dependent morphology evolution of anisotropic hydrogels were prepared by rapidly freeze-drying the reaction solutions at different time. TEM images and corresponding mapping profiles were measured on a JEM-2100F field-emission transmission electron microscope. The samples of Ag/PAM lamellae for the TEM investigation were obtained by directly dropping reaction solution of the hydrogel after UV irradiation of 1–2 min on the copper grid. The samples of 2D lamellar AgNP assemblies for the TEM investigation were prepared by directly dropping the mixture solution of silver NPs and BACA molecules with the same amounts as those in the preparation of hydrogels, after UV irradiation of 5 min, on the copper grid. The TEM images for investigating the silver NP distribution in the hydrogel was carried out on the sample prepared by ultrasonic treatment of the freeze-dried hydrogel pieces in the ethanol. POM image was measured on a microscope MV3000. The absorption spectra were conducted on the UV-Vis spectrophotometer (UV-2600, SHIMADZU). XPS spectra were recorded on an X-ray photoelectron spectrometer (ESCALab MKII). Raman spectra were carried out on a confocal laser microRaman spectrometer (LABRAM-HR). FT-IR spectra were collected by a Thermo Nicolet 6700 spectrometer at room temperature. The dynamic light scattering (DLS) was performed on a Zetasizer Nano S90. Mechanical tests were carried out using an Instron 5965a testing instrument with the stretch speed of 100 mm/min. Photothermal-induced self-healing was triggered by NIR laser (MDL-H-808) with wavelength of 808 nm. The temperature change under NIR irradiation was monitored by a Fluke Ti400 infrared imager.

## Supplementary information

Supplementary Information

## Data Availability

The data that support the findings of this study are available on reasonable request from the corresponding authors (H.-P.C. or S.-H.Y.).
