# Supplementary material for: Anisotropic and self-healing hydrogels with multi-responsive actuating capability
Source: Nat Commun. 2019 May 17;10:2202. doi: 10.1038/s41467-019-10243-8 (PMC6525195; doi:10.1038/s41467-019-10243-8)
Supplement: Supplementary file 1 — Supplementary Information [file 41467_2019_10243_MOESM1_ESM.pdf]

Supplementary Information

Anisotropic and self-healing hydrogels with multi-responsive actuating capability

Qin et al.

Supplementary Figures

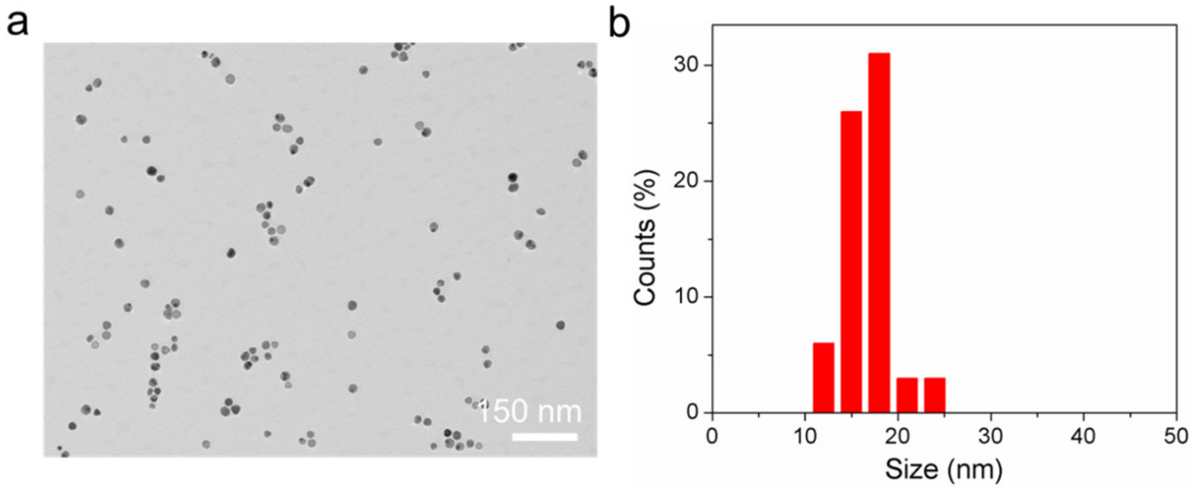

Supplementary Figure 1. (a) TEM image of silver NPs used for preparation of gels. (b) Size distribution histograms of silver NPs, calculated to be 16 ± 2 nm.

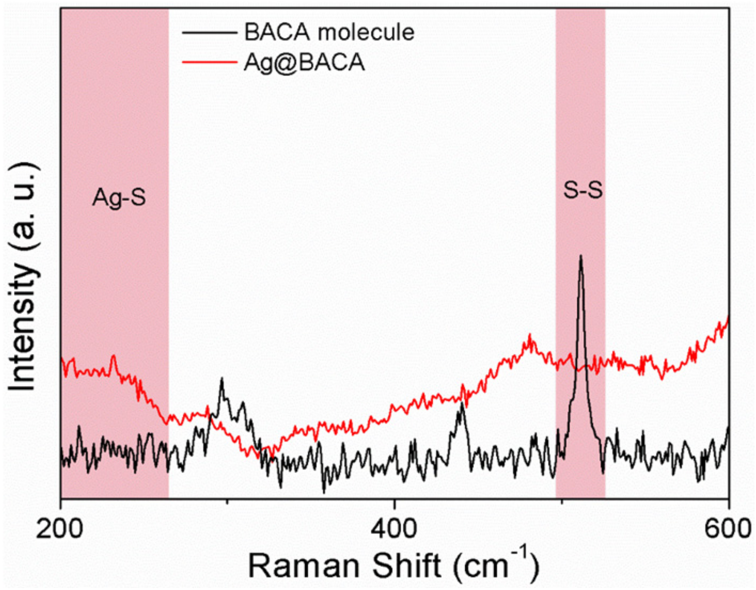

Supplementary Figure 2. Raman spectra of BACA molecule and Ag@BACA composites. The disappearance of 512 cm^{-1} band assigned to S-S stretch and appearance of 200-260 cm^{-1} suggestive of Ag-S stretch indicated the cleavage of S-S bond.

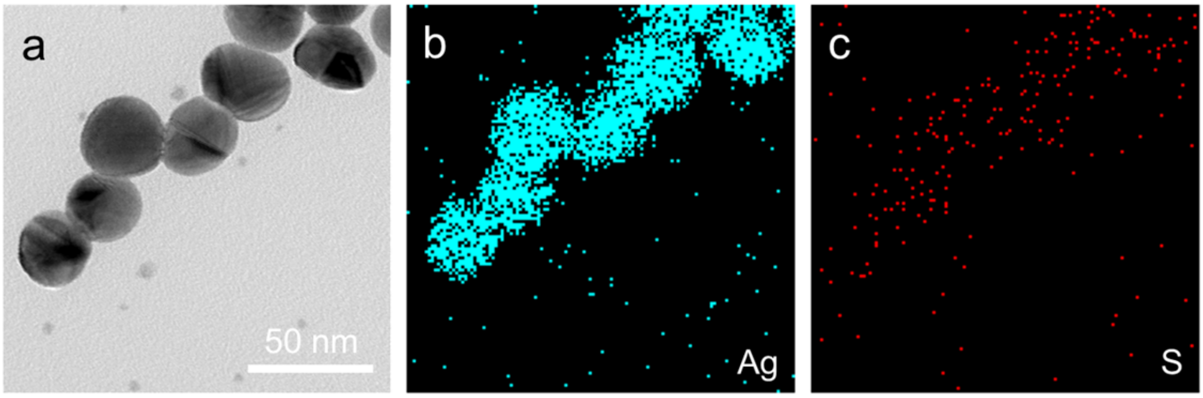

Supplementary Figure 3. (a) TEM image of Ag@BACA nanocomposite and corresponding element mappings of (b) Ag and (c) S, proving the uniform coating of BACA.

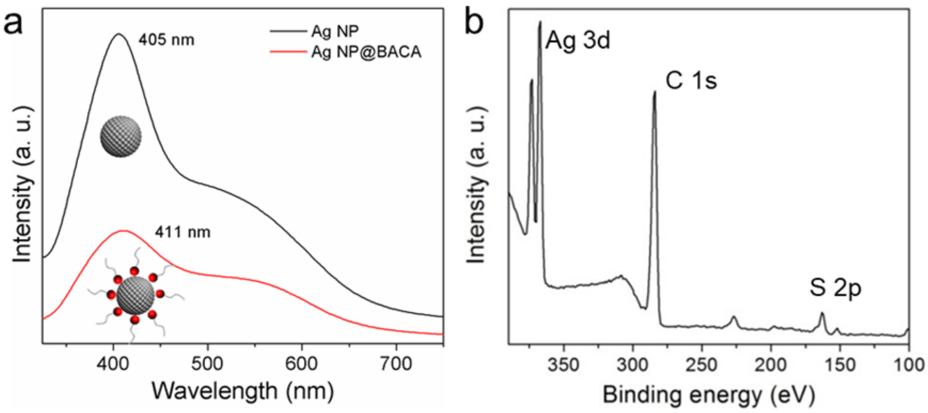

Supplementary Figure 4. (a) UV-vis spectra of silver NPs and Ag@BACA nanocomposite. (b) XPS spectrum of Ag@BACA nanocomposite.

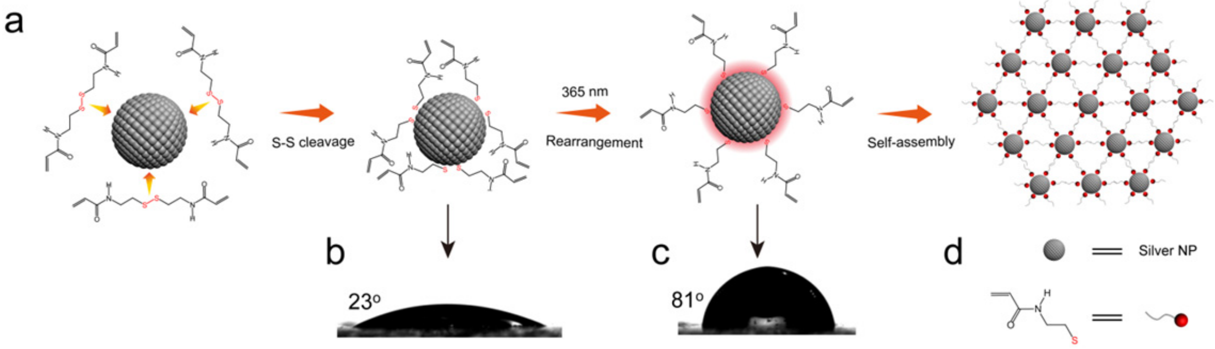

Supplementary Figure 5. (a) Schematic illustration of light-induced self-assembly of Ag NPs. Water contact angle measurements for (b) preliminary adsorption of BACA molecules on Ag NP and (c) nanocomposites after surface rearrangement under UV light irradiation. (d) Scheme of the cleaved BACA.

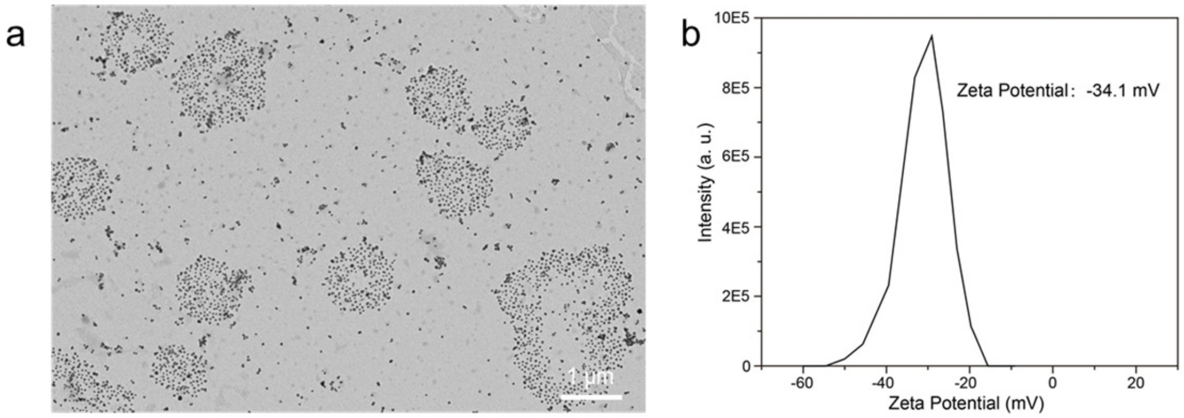

Supplementary Figure 6. (a) SEM image and (b) Zeta potential of the 2D Ag@BACA lamellae assembled from Ag@BACA nanocomposites under the UV irradiation.

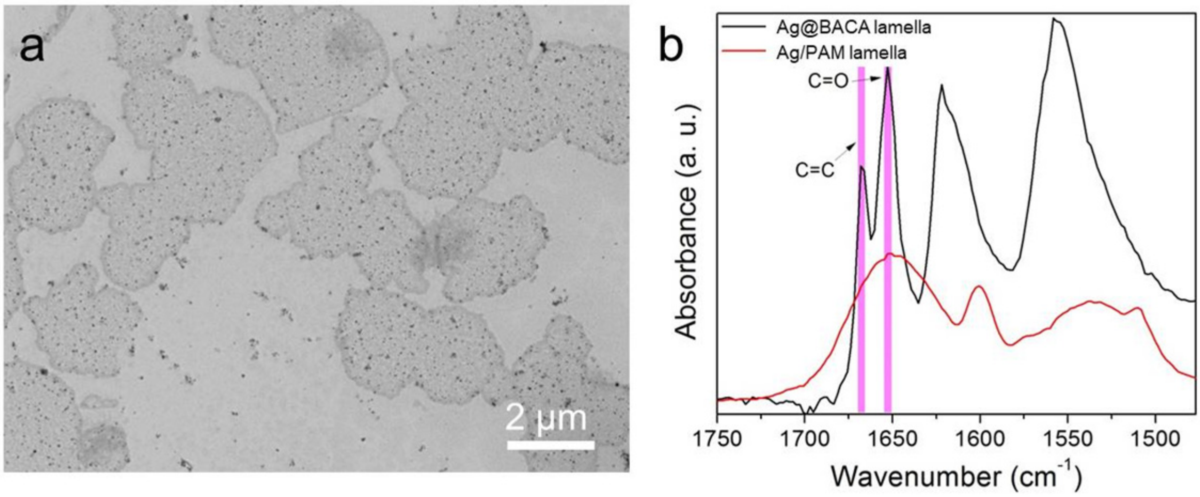

Supplementary Figure 7. (a) TEM image of Ag/PAM lamellae. (b) FT-IR spectra of Ag@BACA lamellae and Ag/PAM lamellae.

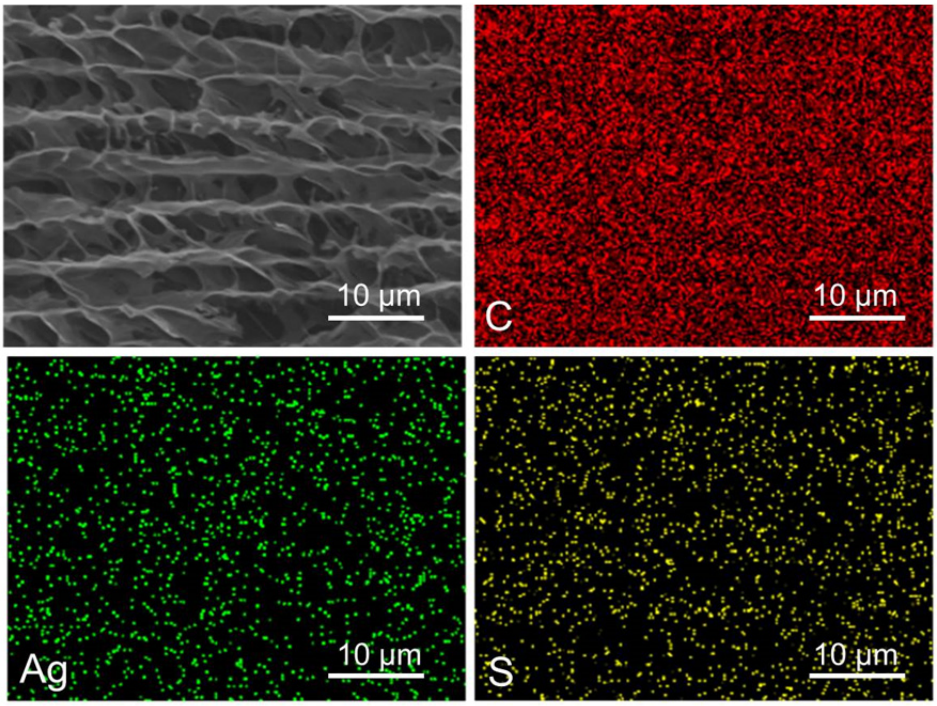

Supplementary Figure 8. SEM image and corresponding element mappings of the freeze-dried SNPP gel.

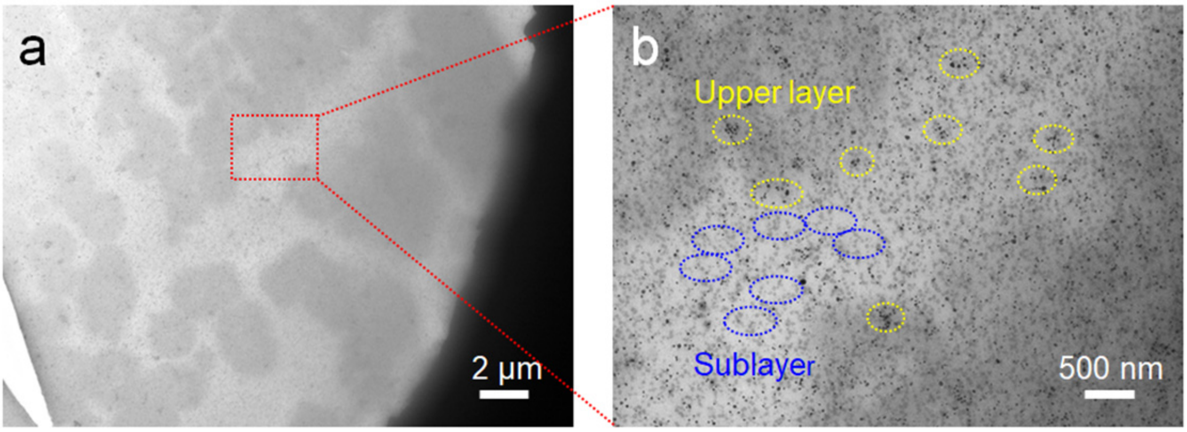

Supplementary Figure 9. (a) Low- and (b) high-magnification TEM images of the hydrogel piece for observation of AgNP distribution.

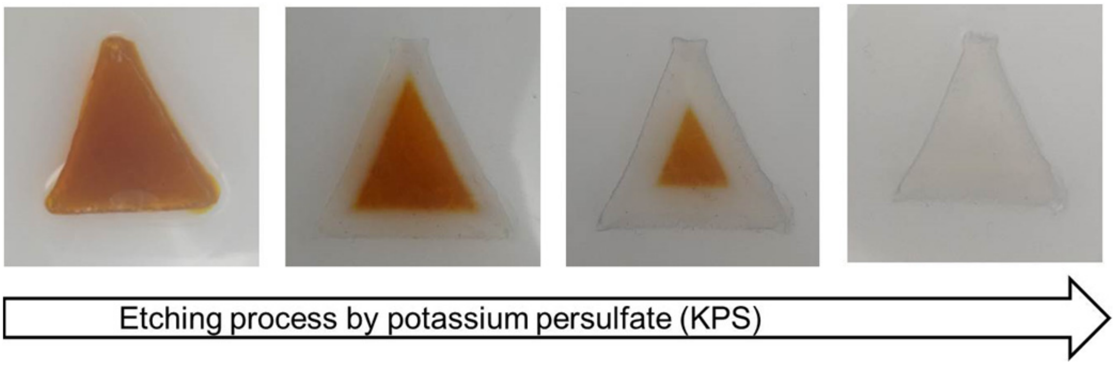

Supplementary Figure 10. Optical images for the etching process of hydrogel by KPS.

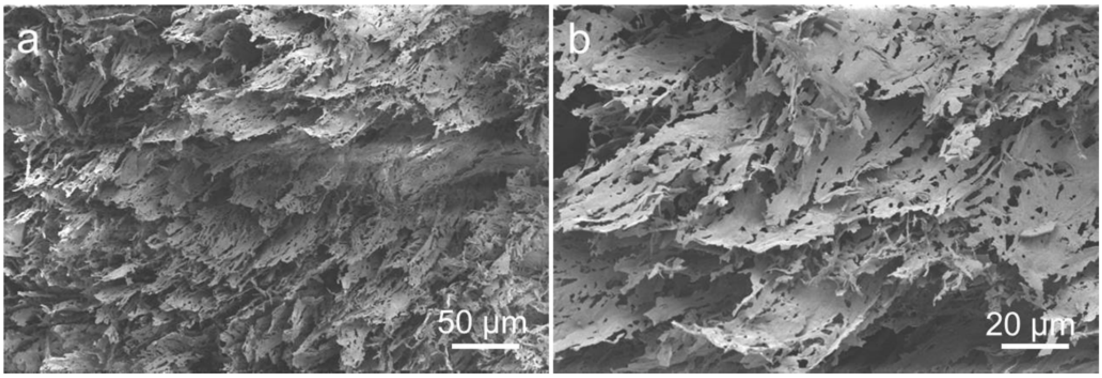

Supplementary Figure 11. SEM images of freeze-dried gels after etching.

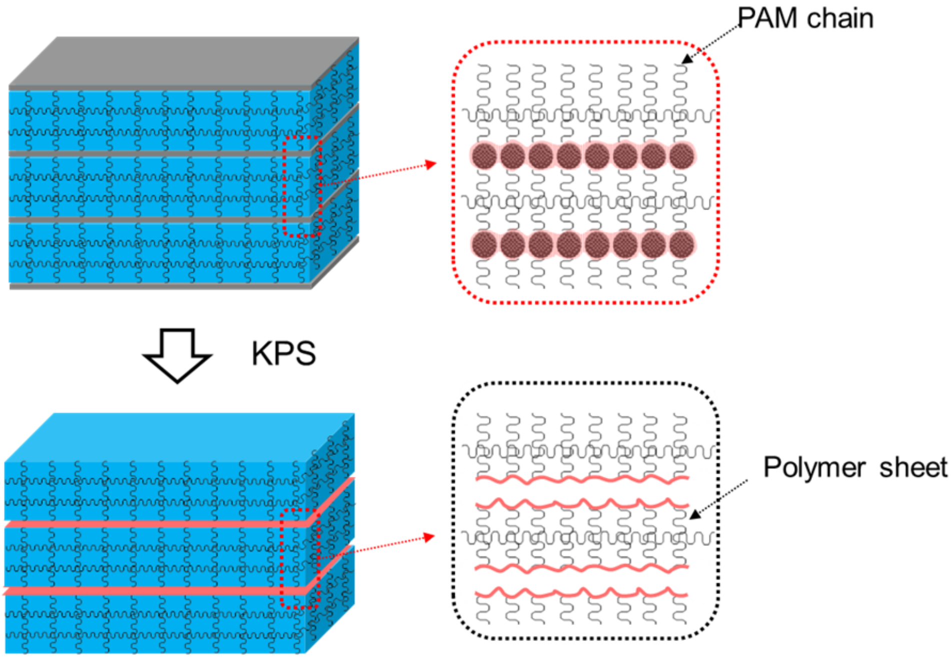

Supplementary Figure 12. Schematic illustration for the etching process of anisotropic hydrogel.

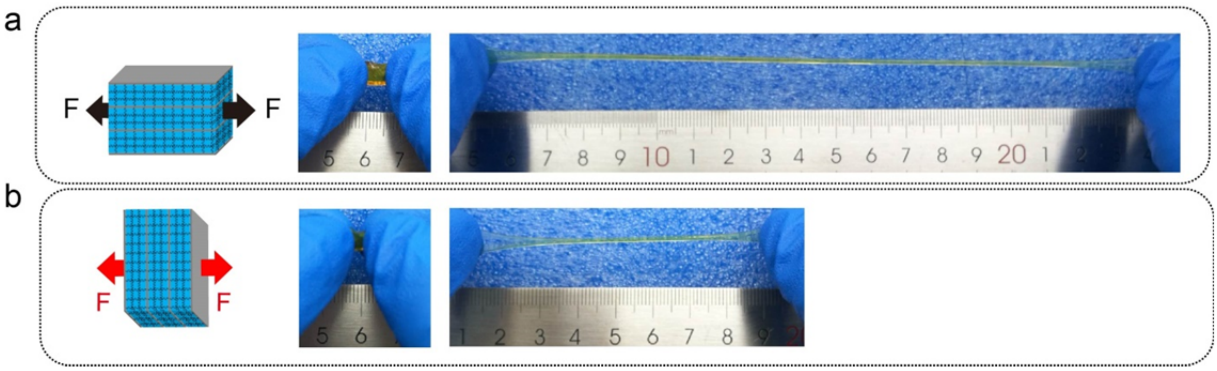

Supplementary Figure 13. Optical images showing the anisotropic stretchabilty of the SNPP hydrogel when stretched in orthogonal directions.

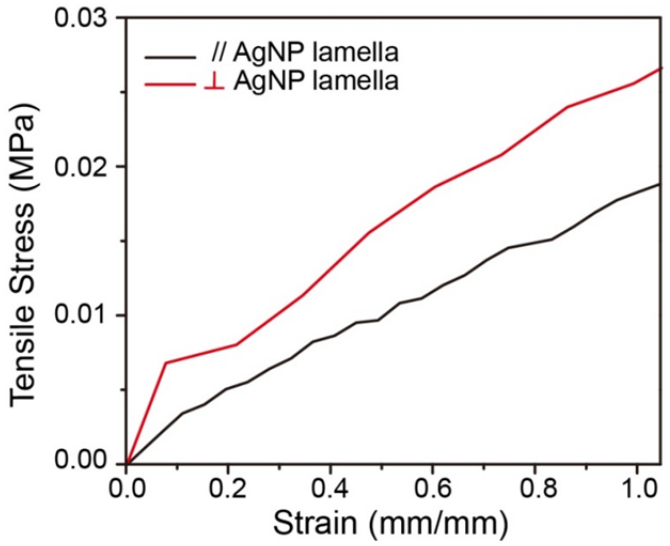

Supplementary Figure 14. Enlarged tensile stress-strain curves of the hydrogel piece with stress applied parallel and perpendicular to the AgNP lamella.

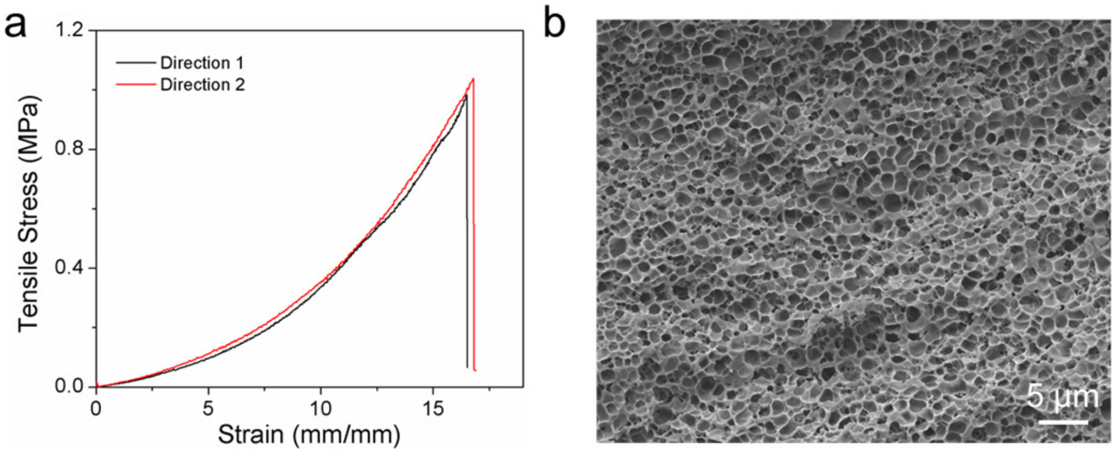

Supplementary Figure 15. Control experiment of hydrogel fabricated without silver NP. (a) Tensile stress-strain curve of hydrogel without Ag NPs when stretched in orthogonal directions. (b) SEM image of free-dried hydrogel network indicating uniform structures.

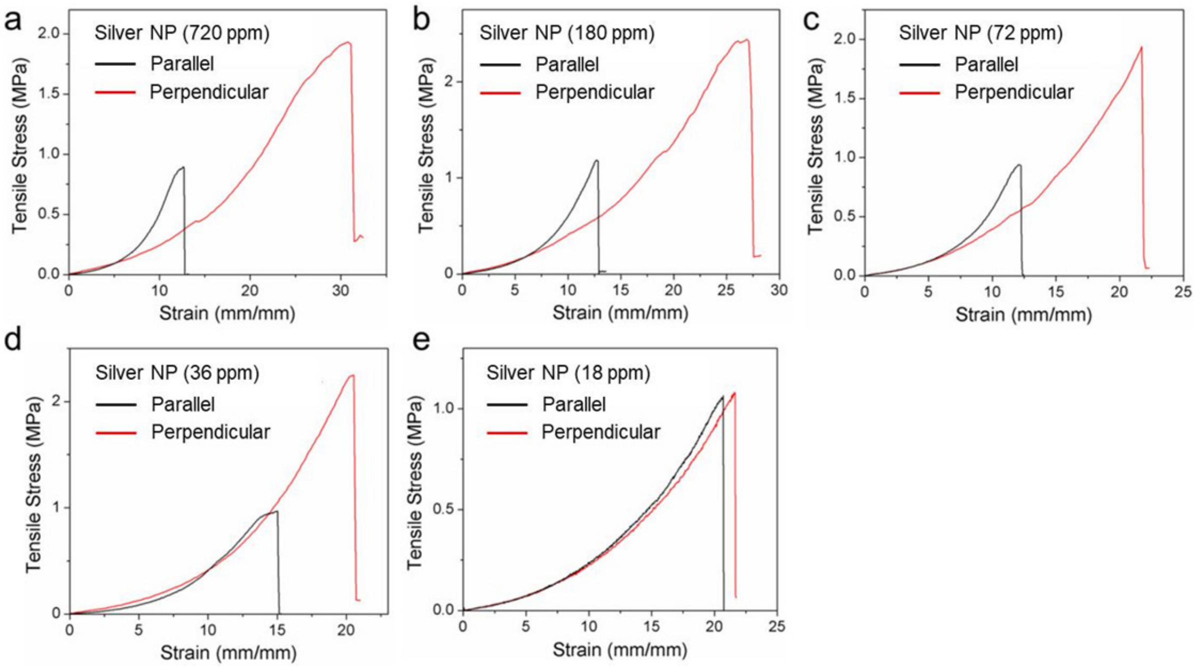

Supplementary Figure 16. Tensile stress-strain curves of hydrogels with different concentrations of silver NPs when stress applied parallel and perpendicular to lamellar structures.

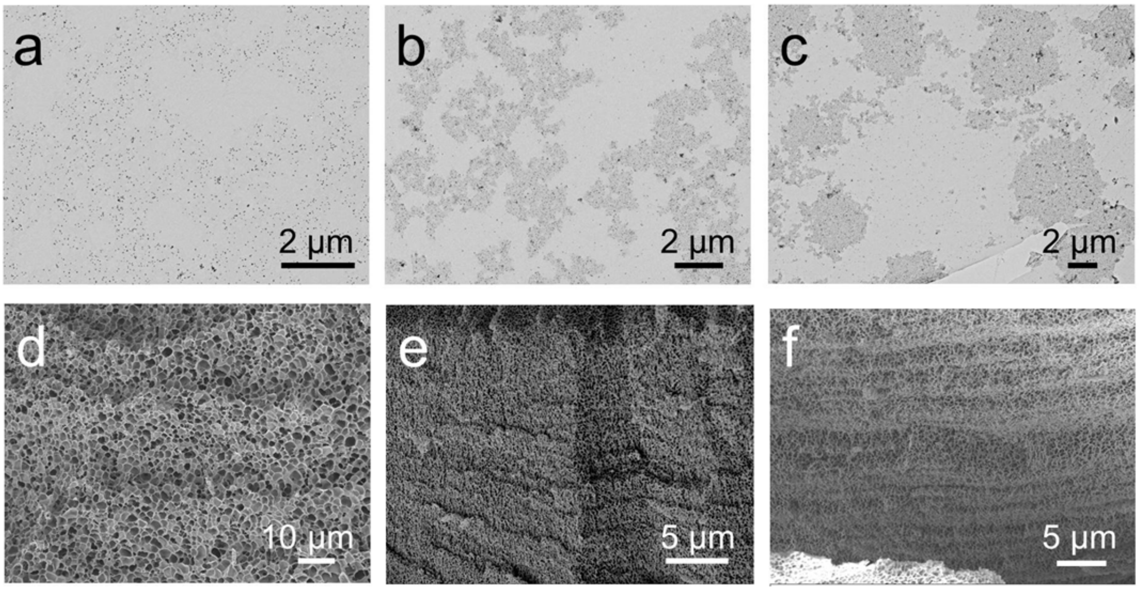

Supplementary Figure 17. TEM images of the assembled AgNP nanostructures and SEM images of freeze-dried gel network obtained with AgNP concentrations of (a, d) 18 ppm, (b, e) 72 ppm and (c, f) 180 ppm, respectively.

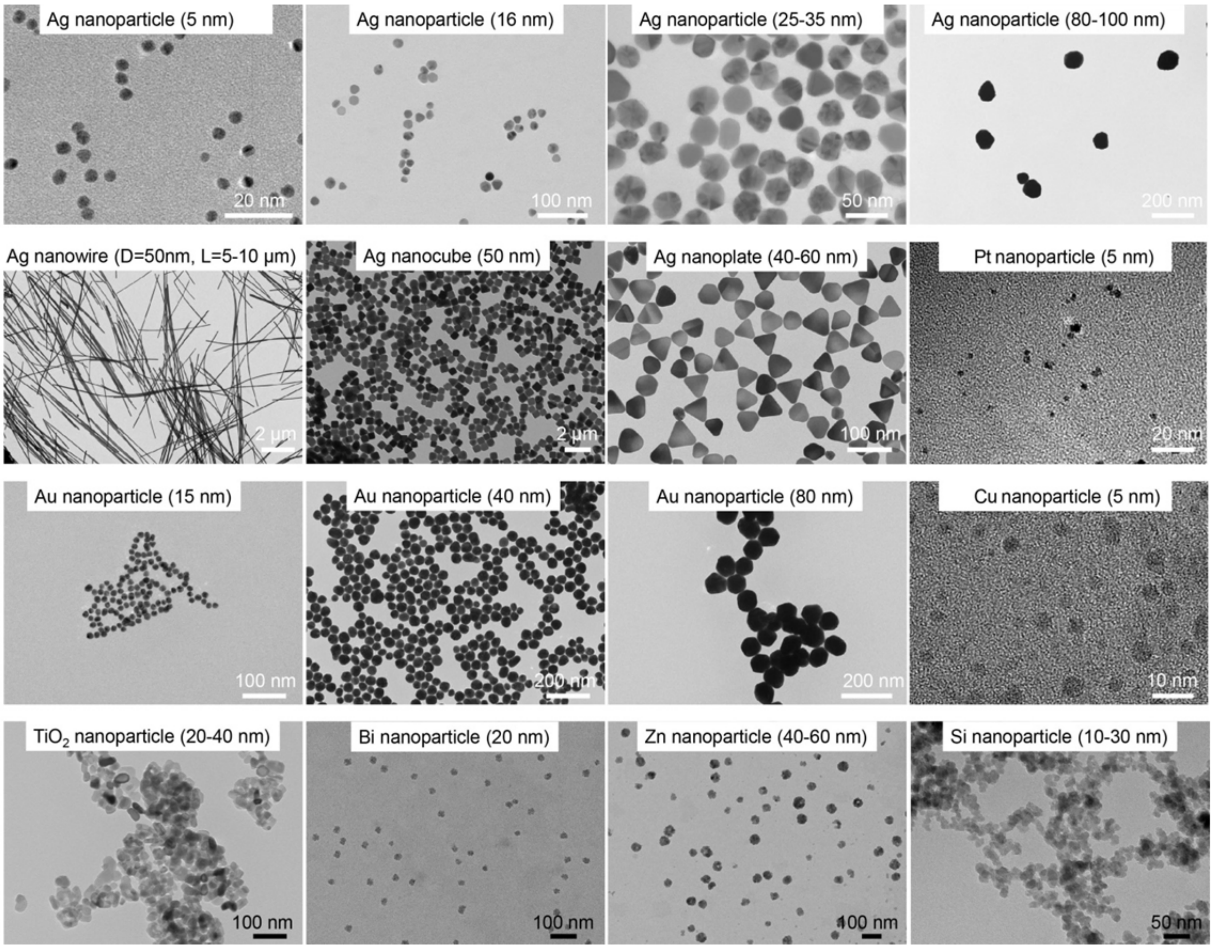

Supplementary Figure 18. TEM images of different nanostructures.

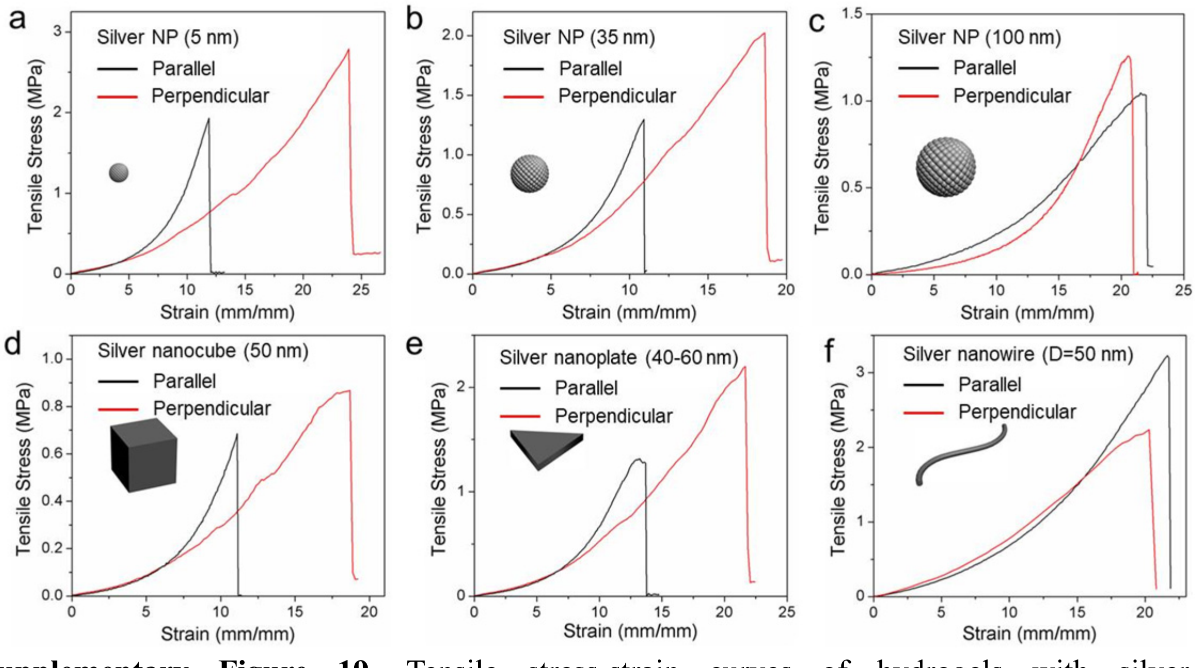

Supplementary Figure 19. Tensile stress-strain curves of hydrogels with silver nanostructures with different sizes and morphologies, when stress applied parallel and perpendicular to lamellar structures.

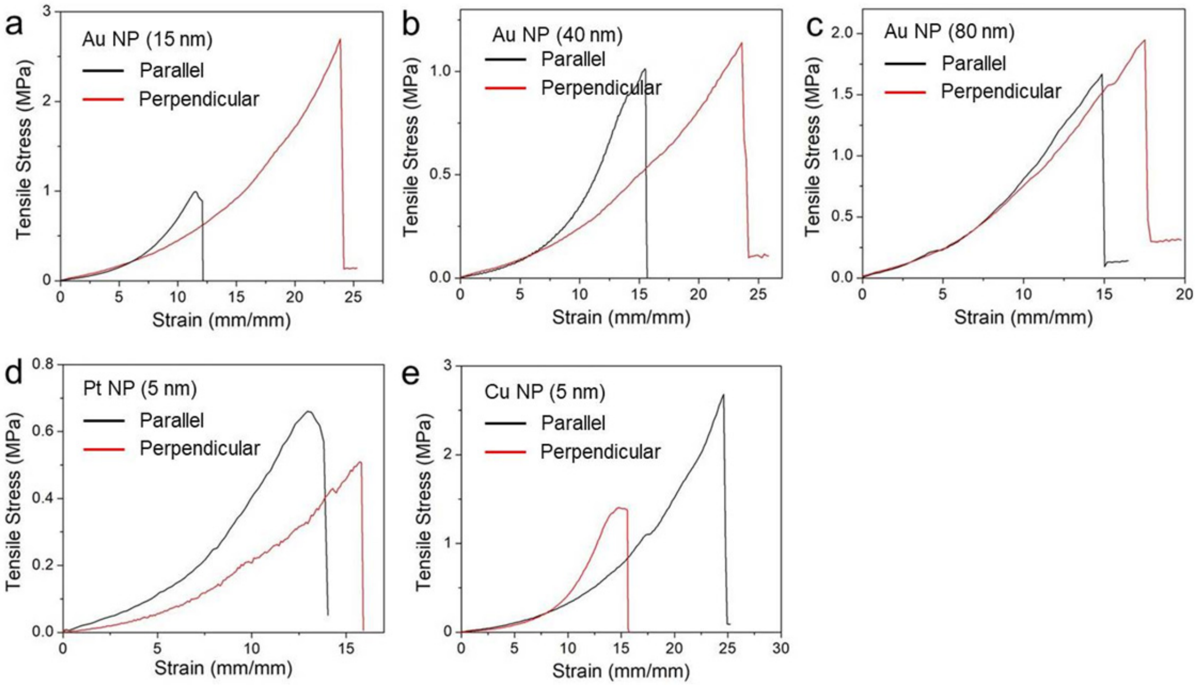

Supplementary Figure 20. Tensile stress-strain curves of hydrogels with precious metal nanoparticles with different sizes, when stress applied parallel and perpendicular to lamellar structures.

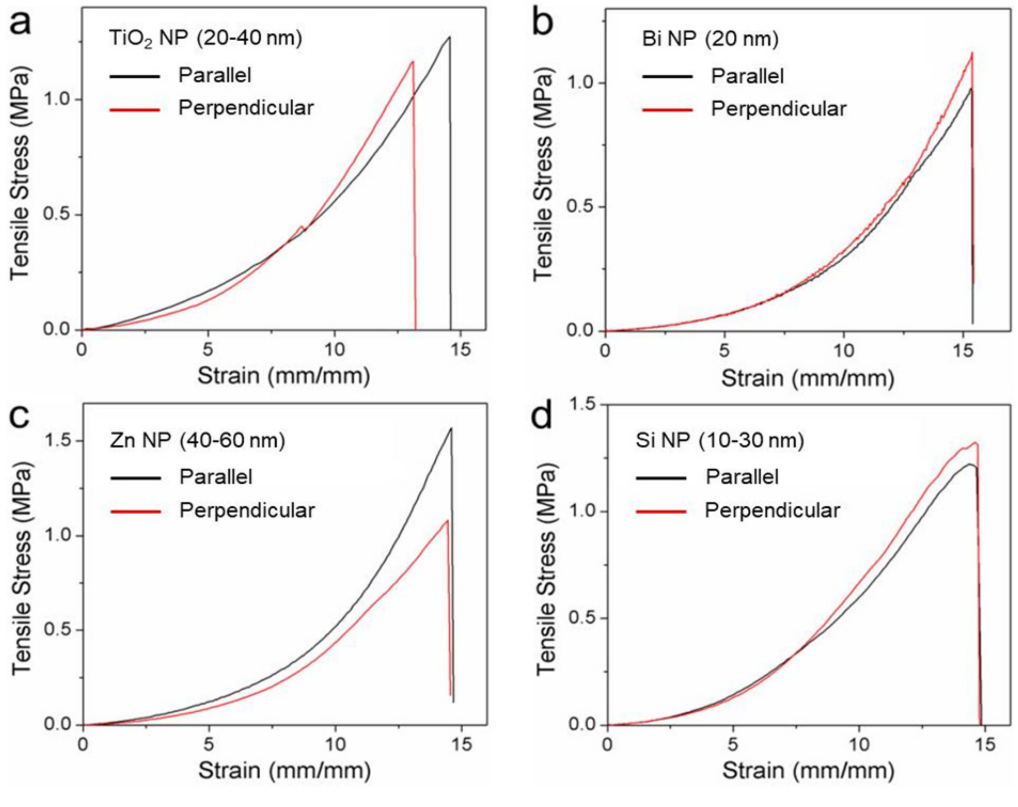

Supplementary Figure 21. Tensile stress-strain curves of hydrogels with nanoparticles of Zn, Bi, Si and TiO_2, when stress applied parallel and perpendicular to lamellar structures.

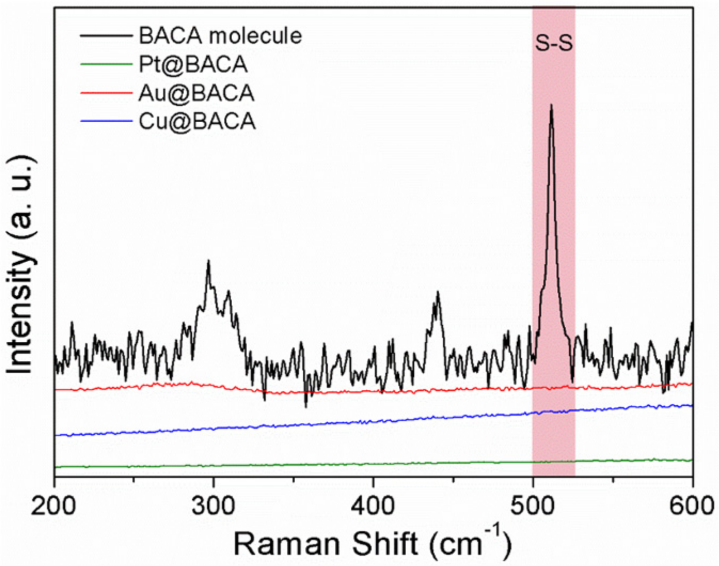

Supplementary Figure 22. Raman spectra of BACA molecule and composites with precious metals, including Pt, Au, and Cu. The disappearance of 512 cm^{-1} band assigned to S-S stretch indicated the cleavage of S-S bond after adsorbed on the surface of precious metal.

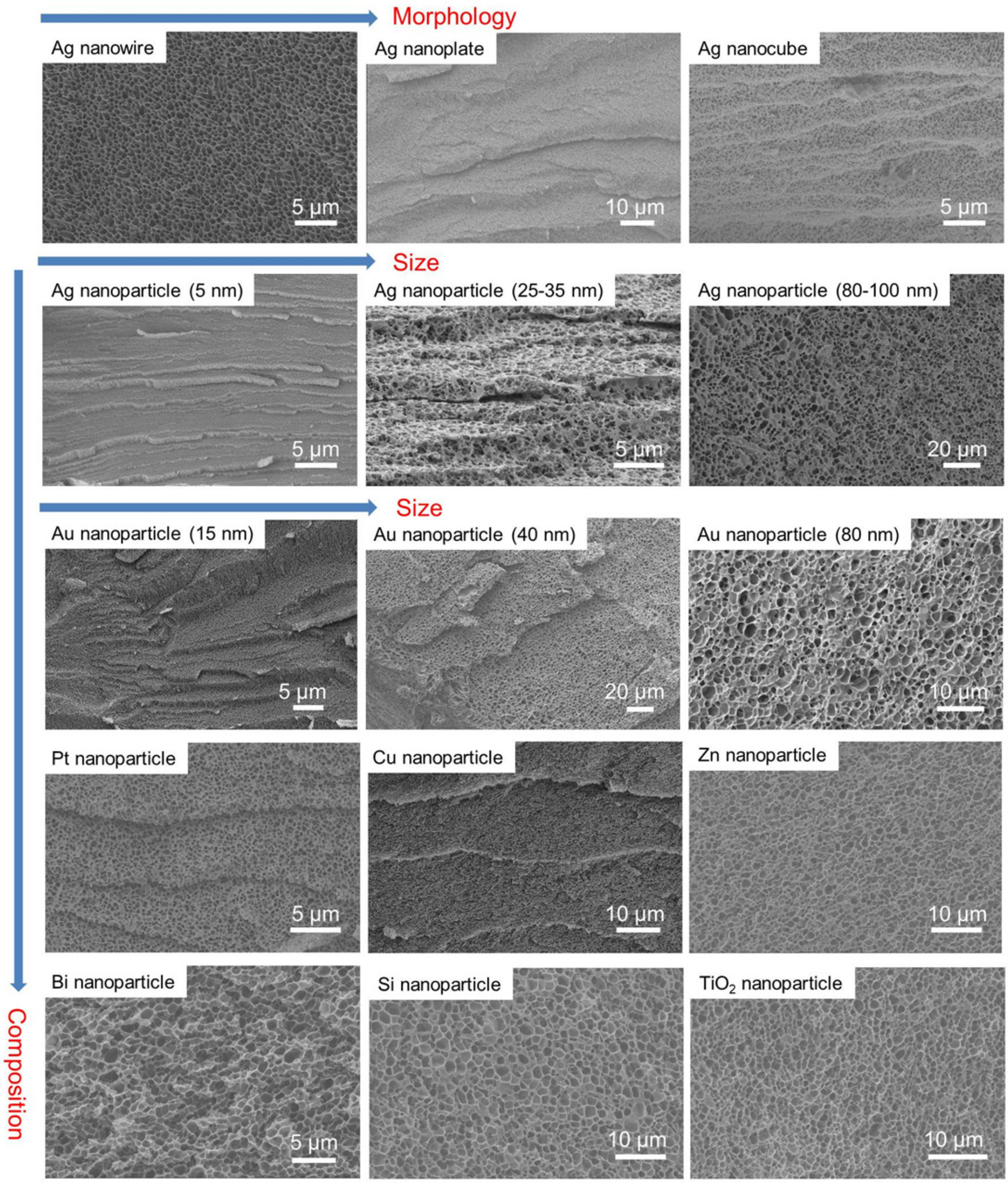

Supplementary Figure 23. SEM images of freeze-dried gel network prepared from 15 nanostructures including precious metals with varying sizes, morphologies and compositions, non-precious metals, nonmetal nanoparticles and metal oxide.

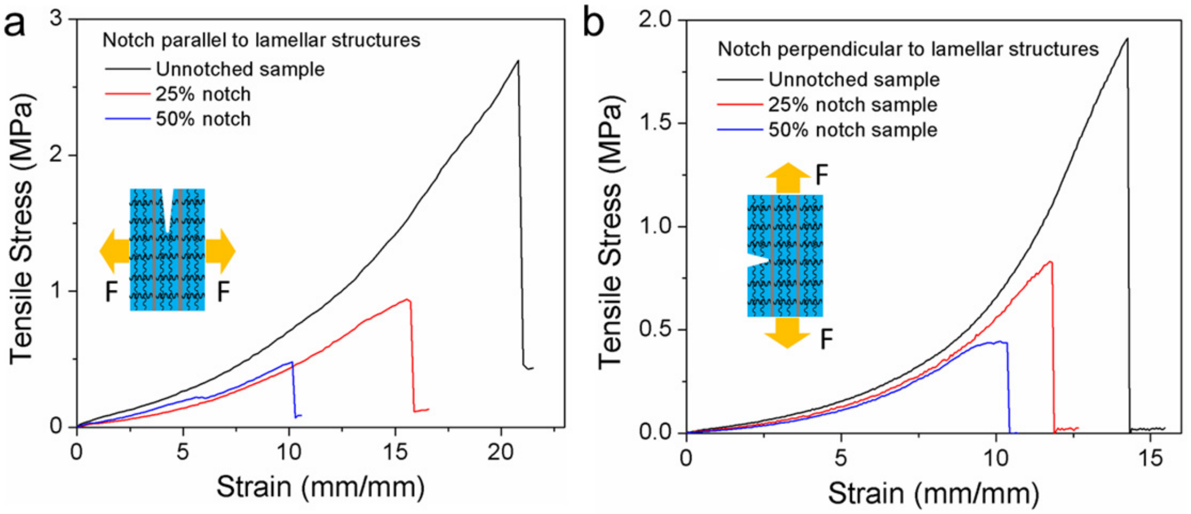

Supplementary Figure 24. Tensile stress-strain curves of hydrogels with different sizes of notch (a) perpendicular and (b) parallel to lamellar structures.

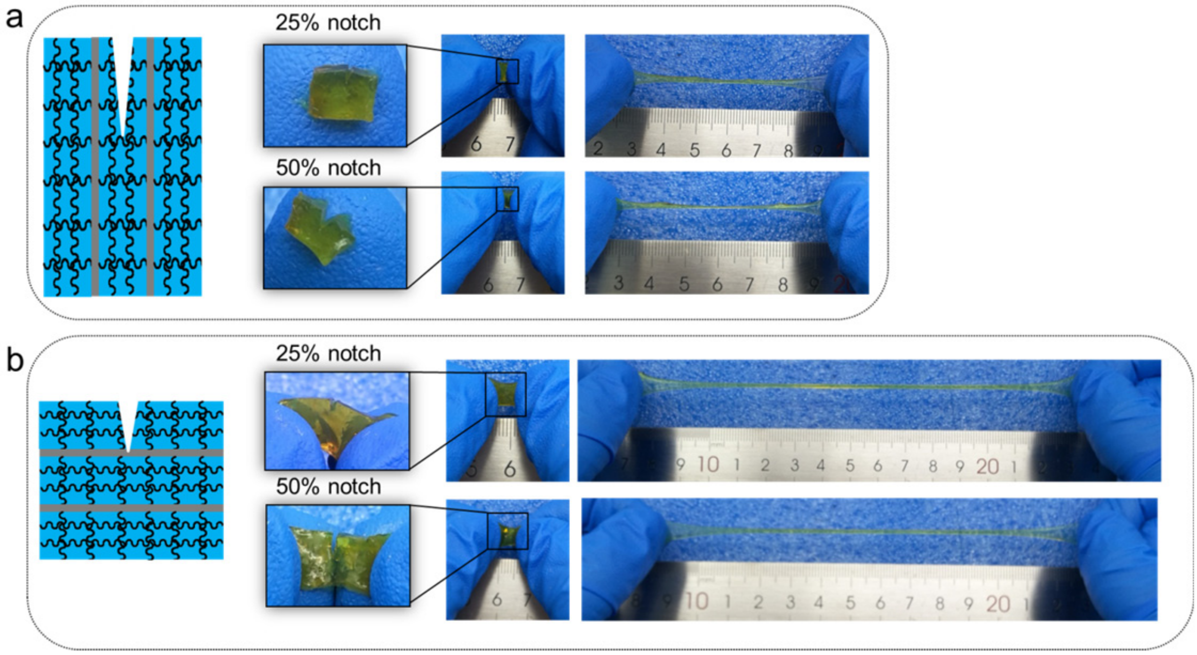

Supplementary Figure 25. Optical images showing the anisotropic notch-intensitivity of hydrogels when notched in orthogonal directions.

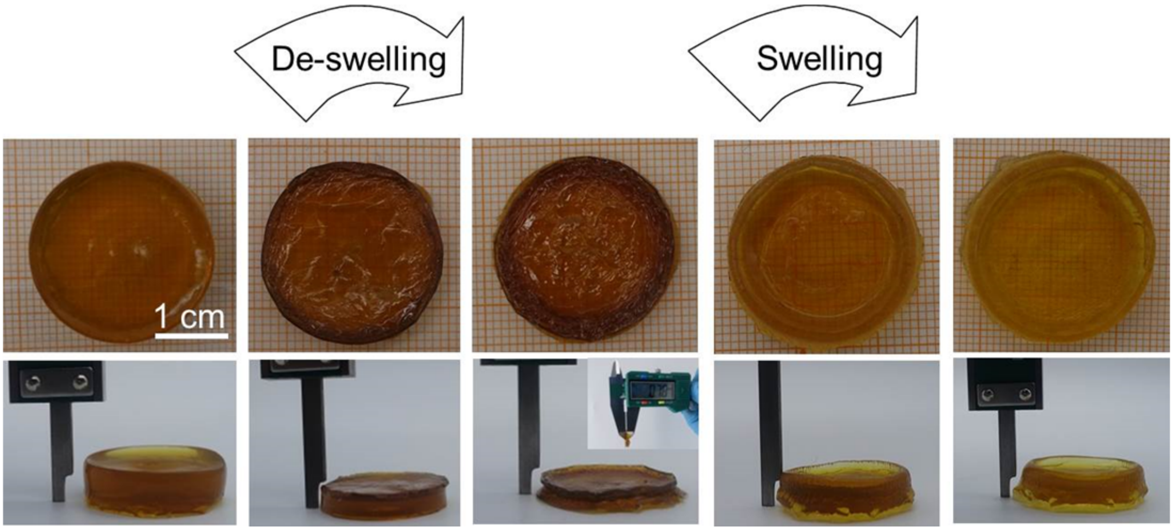

Supplementary Figure 26. Optical images showing the anisotropic de-swelling and swelling behaviors of hydrogels.

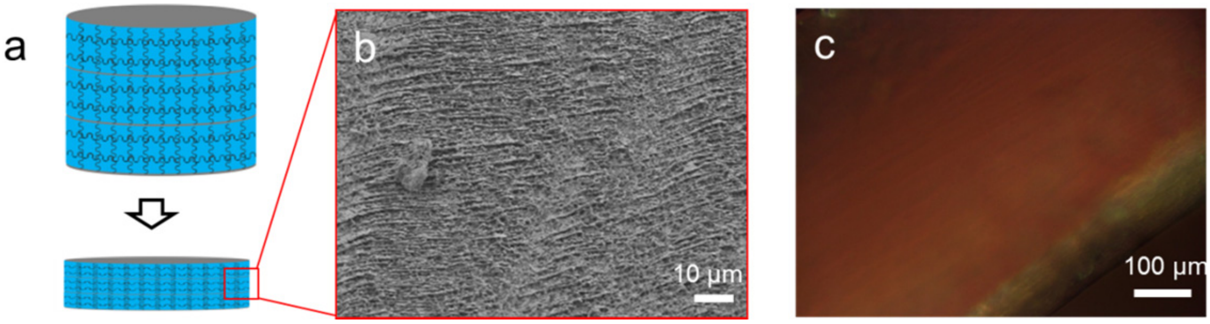

Supplementary Figure 27. (a) Schematic illustration for de-swelling process of anisotropic hydrogel. (b) SEM image of the shrunk gel with densely layered structures. (c) POM image of the shrunk gel viewed parallel to lamellar structures.

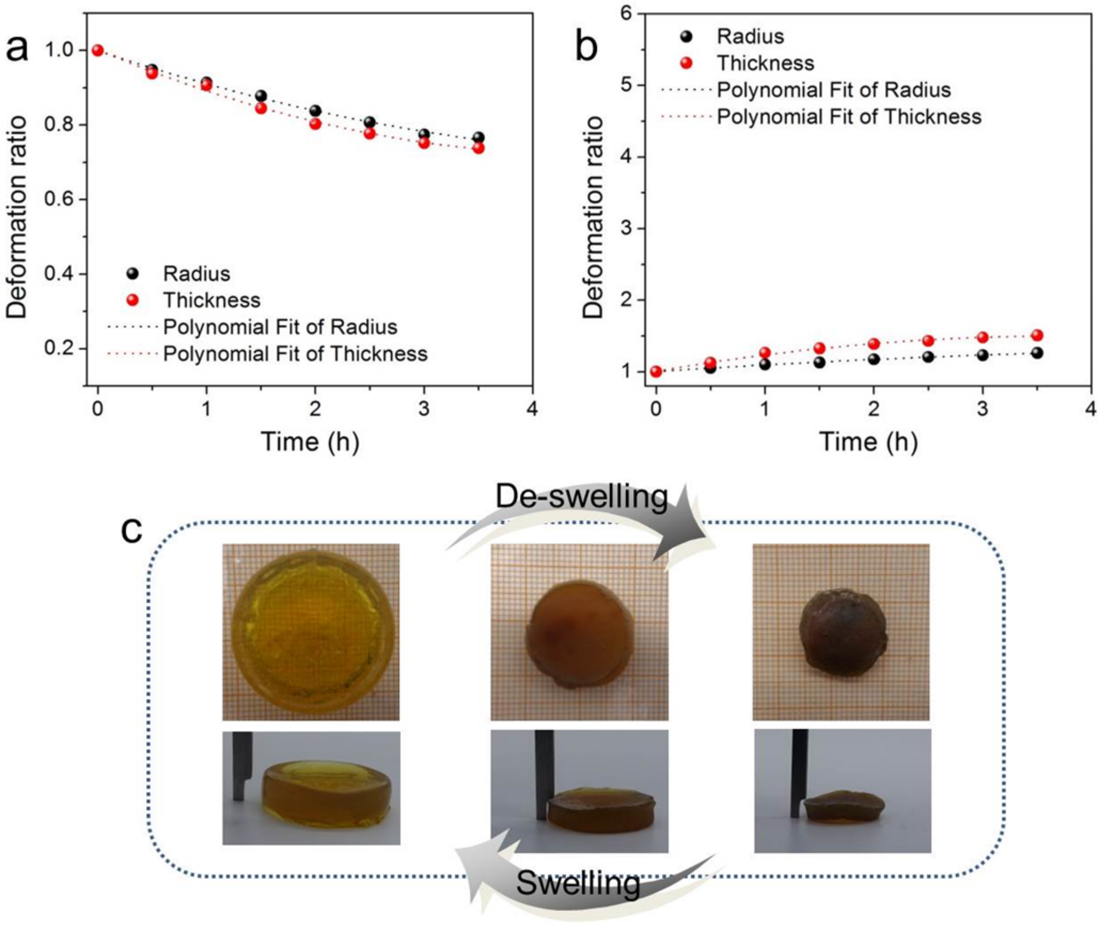

Supplementary Figure 28. Control experiments for de-swelling and swelling performances of conventional hydrogel with silver NPs physically introduced. Plots of deformation ratio of radius and thickness in the de-swelling (a) and swelling (b) processes against time. (c) Optical images of hydrogels during de-swelling and swelling process.

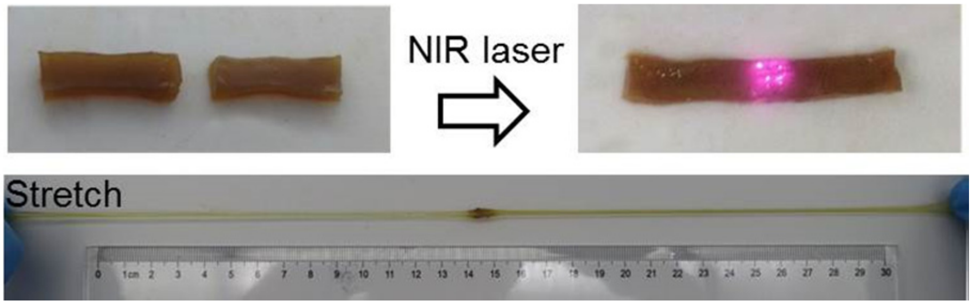

Supplementary Figure 29. Optical images showing the self-healing process between two pieces under NIR laser. The healed sample showed a large deformation without rupture under an external force.

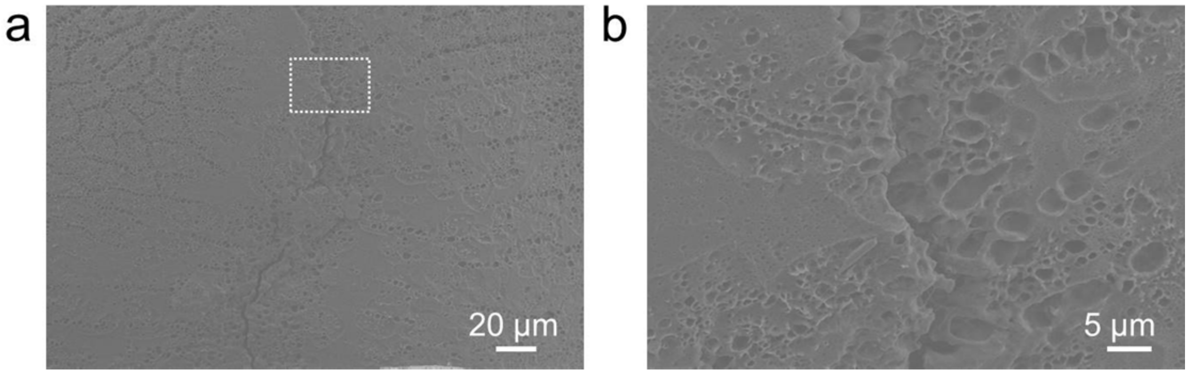

Supplementary Figure 30. (a) Low- and (b) high-magnification SEM images of the healed interface after NIR irradiation.

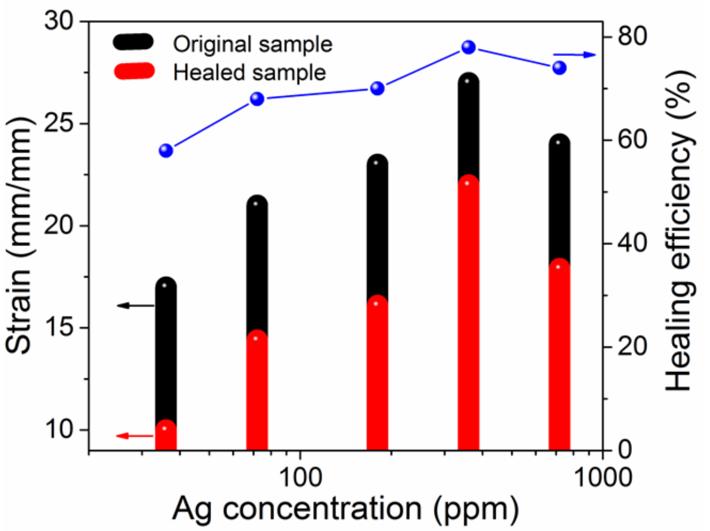

Supplementary Figure 31. Comparison of strain and healing efficiency of SNPP gels with different concentrations of silver when exposed to NIR laser.

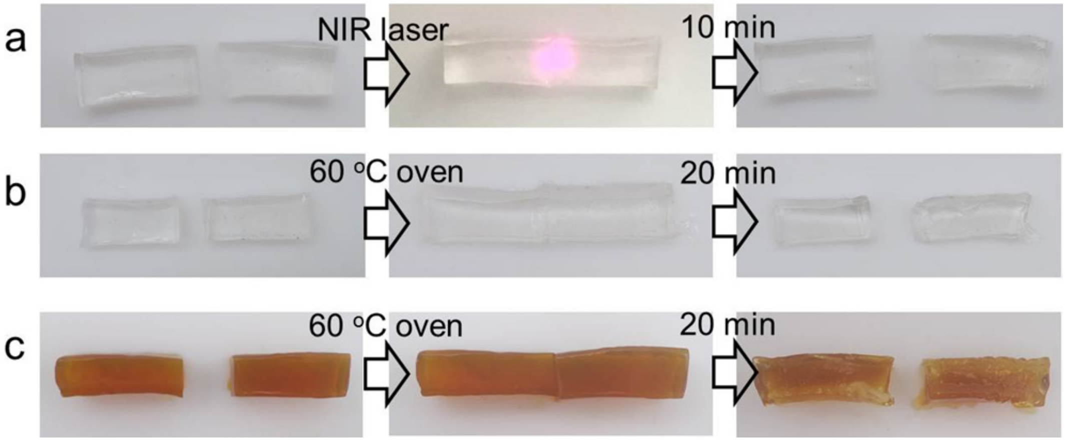

Supplementary Figure 32. Control experiments for verifying the statement on self-healing mechanism. Optical images showing the unhealed hydrogel pieces (SNPP-0) under the irradiation of NIR laser (a) or placed in the oven at 60 °C (b). (c) Optical images of SNPP hydrogel pieces with high Ag NPs placed in the oven at 60 °C for self-healing. Finally, no healing was observed.

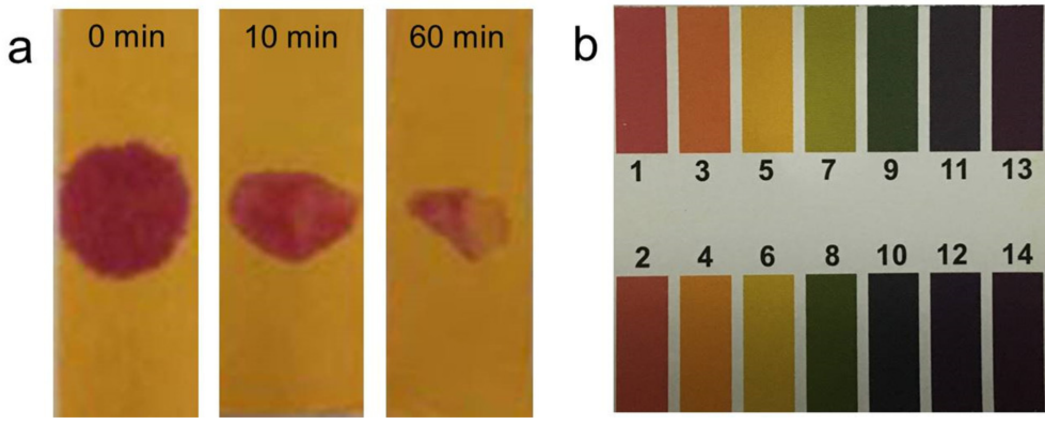

Supplementary Figure 33. (a) Optical images for the pH value change of the hydrogel interface after being brushed with acid solution (1 mol/L) monitored by pH indicator strips with time. (b) Optical image of pH colorimetric card.

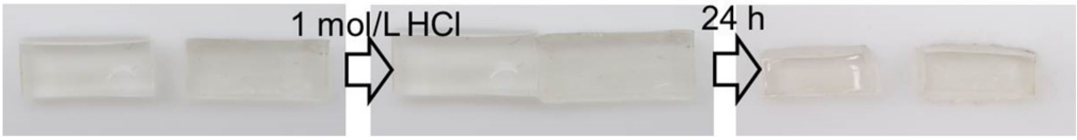

Supplementary Figure 34. Optical images of hydrogel pieces (SNPP-0) during the self-healing process with the aid of HCl solution, and no healing was observed.

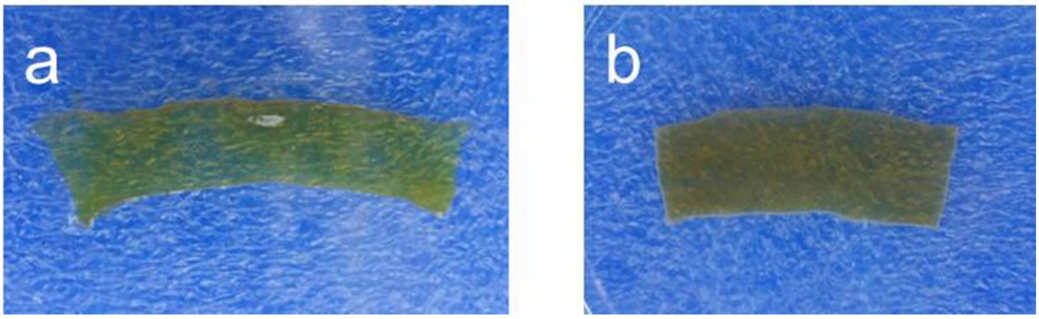

Supplementary Figure 35. Optical images for the deformed gel pieces recovered in water.

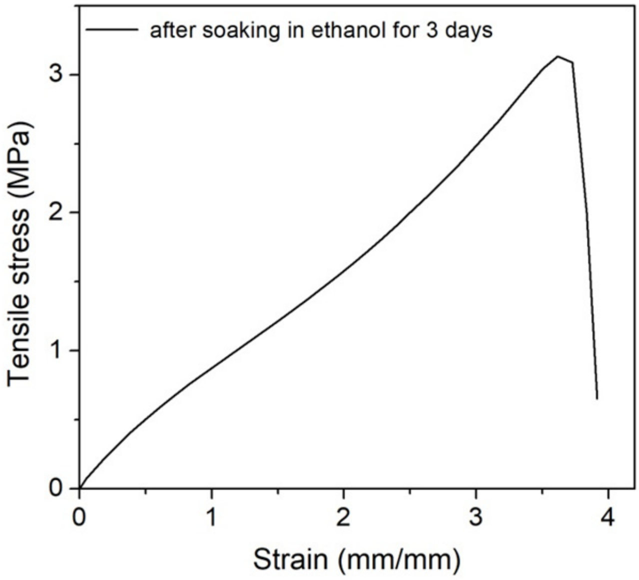

Supplementary Figure 36. Tensile stress-strain curve of hydrogel piece after soaking in ethanol for 3 days.

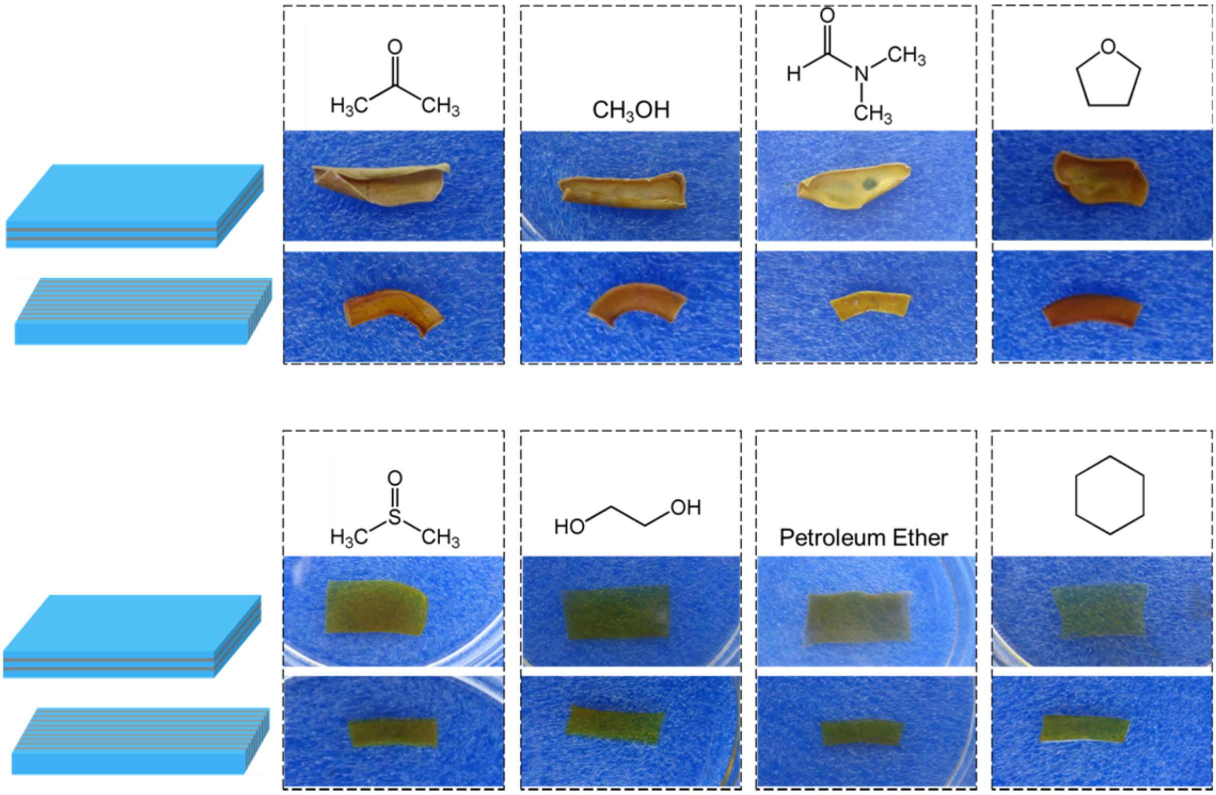

Supplementary Figure 37. Optical images for actuating performance of gel pieces with anisotropic structures when exposed to various solvents. Obvious bending was found when gel pieces placed in the solvents of acetone, methanol, dimethylformamide (DMF), and tetrahydrofuran (THF), while no bending occurred in the solvents of dimethyl sulphoxide (DMSO), ethylene glycol, petroleum ether and cyclohexane.

Supplementary Table 1. Comparison of self-healing performances of SNPP hydrogels with the previously-reported hydrogels.

Substance	Healing mechanism	Healing condition	Healing efficiency	Healing time	Ref.
Clay/G3-binder	Non-covalent bonds	Contact	Horizontally/ vertically stable	--	1
P(NaSS-co-MPTC)	Ionic bonds	Water	99%	24 h	2
PA6ACA	Hydrogen bonds	Low pH solution	66 ± 7%	24 h	3
DMAAm/TiNSs/Laponite	Photolatent modulation	Light ($\lambda > 260$ nm)	Elongation of 500%	20 min	4
βCD-Ad-Fc	Host-guest interactions	Water	52%	24 h	5
DNODN	Hydrogen bonds and aromatic interactions	Contact	Withstand shaking	--	6
CEC-I-OSA-I-ADH	Imine and acylhydrazone bonds	PBS solution	～80%	3 h	7
GNP	RS-Au bonds	NIR laser (808 nm)	96% (Elongation of 2300%)	1 min	8 (Our previous work)
**SNPP**	**RS-Ag bonds**	**NIR laser (808 nm)**	**85% (Elongation of 2100%)**	**10 min**	**This work**

Supplementary Table 2. Comparison of pH-mediated self-healing performances of SNPP hydrogels with the previously-reported hydrogels.

Substance	Healing mechanism	Healing efficiency	Healing time	Ref.
PA6ACA	Hydrogen bonds	66 ± 7% (Elongation of 400%)	24 h	3
DNODN	Hydrogen bonds and aromatic interactions	Withstand shaking	--	6
GO/PAACA	Hydrogen bonds	Withstand stretch	--	9
CEC/PEGDA	Dynamic Schiff base	Withstand holding	3 h	10
PAM-co-PDAAM	Ketone-type acylhydrazone	No splitting	18 h	11
cPEG/BDBA	Boronate-catechnol	Withstand holding	30 s	12
**SNPP**	**RS-Ag bonds**	**45-60% (Elongation of 1100%)**	**3～6 h**	**This work**

Supplementary References

1. Wang, Q. *et al.* High-water-content mouldable hydrogels by mixing clay and a dendritic molecular binder. *Nature* **463**, 339-343 (2010).

2. Sun, T. L. *et al.* Physical hydrogels composed of polyampholytes demonstrate high toughness and viscoelasticity. *Nat. Mater.* **12**, 932-937 (2013).

3. Phadke, A. *et al.* Rapid self-healing hydrogels. *P. Natl. Acad. Sci. USA* **109**, 4383-4388 (2012).

4 Liu, M. *et al.* Photolatently modulable hydrogels using unilamellar titania nanosheets as photocatalytic crosslinkers. *Nat. Commun.* **4**, 2029 (2013).

5. Miyamae, K., Nakahata, M., Takashima, Y., Harada, A. Self-healing, expansion-contraction, and shape-memory properties of a preorganized supramolecular hydrogel through host-guest interactions. *Angew. Chem. Int. Ed.* **54**, 8984-8987 (2015).

6. Li, L. *et al.* Novel mussel-inspired injectable self-healing hydrogel with anti-biofouling property. *Adv. Mater.* **27**, 1294-1299 (2015).

7. Wei, Z. *et al.* Novel biocompatible polysaccharide-based self-healing hydrogel. *Adv. Funct. Mater.* **25**, 1352-1359 (2015).

8. Qin, H. *et al.* Dynamic Au-thiolate interaction induced rapid self-healing nanocomposite hydrogels with remarkable mechanical behaviors. *Chem* **3**, 691-705 (2017).

9. Cong, H.-P, Wang, P, Yu, S.-H. Stretchable and self-healing graphene oxide–polymer composite hydrogels: a dual-network design. *Chem. Mater.* **25**, 3357-3362 (2013).

10. Qu, J., Zhao, X., Ma, P. X., Guo, B. pH-responsive self-healing injectable hydrogel based on N-carboxyethyl chitosan for hepatocellular carcinoma therapy. *Acta Biomater.* **58**, 168-180

(2017).

11. Guo, Z. *et al.* pH-Switchable and self-healable hydrogels based on ketone type acylhydrazone dynamic covalent bonds. *Soft Matter* **13**, 7371-7380 (2017).

12. He, L., Fullenkamp, D. E., Rivera, J. G., Messersmith, P. B. pH responsive self-healing hydrogels formed by boronate-catechol complexation. *Chem. Commun.* **47**, 7497-7499 (2011).
